# Immune checkpoint inhibitors efficacy across solid cancers and the utility of PD-L1 as a biomarker of response: a systematic review and meta-analysis

**DOI:** 10.3389/fmed.2023.1192762

**Published:** 2023-05-12

**Authors:** Timothy S. Fitzsimmons, Niharika Singh, Thomas D. J. Walker, Claire Newton, Dafydd G. R. Evans, Emma J. Crosbie, Neil A. J. Ryan

**Affiliations:** ^1^Clinical Medical School, University of Bristol, Bristol, United Kingdom; ^2^Division of Cancer Sciences, St Mary’s Hospital, Faculty of Biology, Medicine and Health, University of Manchester, Manchester, United Kingdom; ^3^Department of Obstetrics and Gynaecology, St Michaels Hospital, Bristol, United Kingdom; ^4^Division of Evolution and Genomic Medicine, St Mary’s Hospital, University of Manchester, Manchester, United Kingdom; ^5^The College of Medicine and Veterinary Medicine, University of Edinburgh, Edinburgh, United Kingdom; ^6^Department of Gynaecology Oncology, Royal Infirmary of Edinburgh, Edinburgh, United Kingdom

**Keywords:** pan-cancer therapy, checkpoint immune blockade antibodies, PDL1, mismatch repair, oncology, immunotherapy

## Abstract

**Background:**

Immune checkpoint inhibitors (ICPI) are a tumor agnostic treatment. However, trials of their use have been site specific. Here we summarize the trial data and explore the utility of programmed death-ligand 1 (PD-L1) expression as a biomarker to direct their pan-cancer use.

**Method:**

A systematic review of literature, following PRISMA guidelines, was performed. Medline, Embase, Cochrane CENTRAL, NHS Health and Technology, and Web of Science were searched from their conception to June 2022 limited to the English language. The search terms and method were devised by a specialist medical librarian. Studies were limited to adults with solid cancers (excluding melanomas) treated with ICPIs. Only phase III randomized control trials (RCT) were included. The primary outcome was overall survival and secondary outcomes were progression free survival, PD-L1 expression, quality of life outcomes and adverse event data. Where present in eligible clinical trials, hazard ratios (HR), risk ratios (RR), standard error (SE) and 95% confidence intervals (CI) were extracted or calculated. Heterogeneity across studies was described with the use of an *I^2^* score (Low: 25, 50%: moderate, 75% low heterogeneity). HR pools inverse variance methods were adopted by Random Effects (RE). Means were standardized across any heterogenous scale limits.

**Results:**

In total 46,510 participants were included in the meta-analysis. Overall, meta-analysis favored the use of ICPIs with an overall survival (OS) HR of 0.74 (95% CI 0.71 to 0.78). Lung cancers showed the most benefit in OS [HR 0.72 (95% 0.66–0.78)] followed by head and neck cancers [HR 0.75 (95% CI 0.66–0.84)] and gastroesophageal junction cancers [HR 0.75 (95% CI 0.61–0.92)]. ICPIs seem to be efficacious at both primary presentation and recurrence [OS HR 0.73 (95% CI 0.68–0.77)] vs. [OS HR 0.79 (95% CI 0.72 to 0.87)] respectively. Interestingly, subgroup analysis comparing studies in which most cancers demonstrated PD-L1 expression vs. those studies in which a minority of cancer demonstrated PD-L1 expression reported similar effect of ICPI use on OS; oddly the data favored ICPI use in studies with a minority of PD-L1 expression. Specifically, studies with minority PD-L1 expression had an HR 0.73 (95% CI 0.68–0.78) vs. studies with majority PD-L1 expression HR 0.76 (95% CI 0.70–0.84). This was maintained even when studies exploring the same cancer site were directly compared. Subgroup analysis was performed comparing the impact on OS subdivided by the specific ICPI used. Where meta-analysis was performed, Nivolumab led to the greatest impact [HR 0.70 (95% CI 0.64–0.77)] with Avelumab failing to reach significance [HR 0.93 (95% CI 0.80–1.06)]. However, overall heterogenicity was high (*I*^2^ = 95%). Finally, the use of ICPIs led to an improved side effect profile when compared with standard chemotherapy [RR 0.85 (95% CI 0.73–0.98)].

**Conclusion:**

ICPIs improve survival outcomes in all cancer types. These effects are seen in the primary, recurrent, chemotherapy sensitive, chemotherapy resistant disease. These data support their use as a tumor agnostic therapy. Furthermore, they are well tolerated. However, PD-L1 as a biomarker for the targeting of ICPI use seems problematic. Other biomarkers such as mismatch repair or tumor mutational burden should be explored in randomized trials. In addition, there are still limited trials looking at ICPI use outside of lung cancer.

## Background

Cancer is the second leading cause of death in the world; only behind cardiovascular disease (1). Despite advancements in treatments, the mortality rate for many cancers remains high (2). For patients with advanced cancer, chemotherapy and radiotherapy are the primary treatment options. However, due to the systemic nature of chemotherapy, there have been issues with toxic side effects as well as drug resistance. Targeted therapies are therefore of intrinsic value as they seek to reduce treatment toxicity and resistance (3). One such targeted therapy are immune checkpoint inhibitors (ICPIs).

Immune checkpoint inhibitors are a class of treatment that exploit a common mechanism of cancer immune escape: the programmed death-1/programmed death ligand (PD-1/PD-L1) ligand/receptor interaction (4). Indeed, cancers that arise due to a defective mismatch repair system commonly exploit the PD-1/PD-L1 pathway (5). Cancers which overexpress PD-L1 inhibit cytotoxic T cells (6). These deactivated T cells remain in the tumor microenvironment as they are continuously recruited through the production of cancer related neo-antigens (7). ICPIs are monoclonal antibodies that act to block the PD-1/PD-L1 axis and reverse the induced T cell exhaustion to prevent cancer immune escape (8). They lead to a re-activation of the recruited tumor associated lymphocytes and tumor containment or eradication.

ICPIs have been trialed in numerous cancer sites with generally encouraging results (9). They were the first class of drug to receive approval from the Federal Drug Agency (FDA) based on a molecular characteristic within the tumor (microsatellite instability (MSI) or high mutational burden (HMB)) as opposed to the anatomical cancer site (10, 11). This approval was based on pooled data of single arm cohorts from trials as no meta-analysis existed (12). However to date, most trials have used PD-L1 as a biomarker for ICPI use and not MSI or HMB (13).

The use of PD-L1 as a biomarker for ICPI is based on its use in the initial trials done in melanomas (14). This is despite the original study that explored ICPI use in melanoma reporting that PD-L1 did not predict those in whom ICPI would lead to significant improved survival (15). There remains limited data synthesis as to the utility of PD-L1 as a biomarker of ICPIs effectiveness across all cancer sites. These data are important given the clinical application of ICPIs is not based on cancer site but tumoral biomarkers. This analysis is also prudent given the increasing use of ICPIs across multiple cancer sites, despite a relatively limited evidence base for their use in that specific cancer site (16). If pan-cancer analysis supported their application based on the expression of a biomarker, clinicians could be more confident in trialing ICPIs in these lesser studied cancer types based on the molecular profile of the cancer. In addition, if PD-L1 expression proved to be an accurate predictor of ICPIs treatment efficacy across multiple cancer sites, its sustained use as such a biomarker in trials would become clinically meaningful.

The aim of this study was to perform a systematic review and meta-analysis to synthesis the existing trial data evaluating the ICPIs use in all solid cancer types. Our hope was to provide evidence as to their cross-cancer utility and help inform their current application based on molecular characteristics as opposed to anatomical cancer site. In addition, we will explore the utility of PD-L1 as a biomarker of ICPI treatment efficacy.

## Method

### Search strategy and study identification

A systematic review of literature, following PRISMA guidelines, was performed (17). Medline, Embase, Cochrane CENTRAL, NHS Health and Technology, and Web of Science were searched from their conception to May 2022. The search terms and method were devised by a specialist medical librarian. In addition, we searched for non-published trial data via www.controlled-trials.com/rct and www.cancer.gov/clinicaltrials. Initial search results were supplemented by citation searching. Non-electronic and grey literature were excluded. The search methods are detailed in Supplementary material section 1.

### Selection criteria

The protocol for this systematic review was preregistered with the PROSPERO database registration (ref: CRD420202219410). Only studies published in English were included. Studies were limited to adults with solid cancers (excluding melanomas) treated with ICPIs. Melanoma was excluded due to its exceptionally high expression of PD-L1 and the extensive evidence indicating ICPI therapeutic efficacy along with standardized PD-L1 immunohistochemistry protocols (18). These factors make it a distinct clinical entity that would be problematic to include in a pan-cancer meta-analysis. Only phase III randomized control trials were included. The primary outcome was overall survival with secondary outcomes being progression free survival, PD-L1 expression, quality of life outcomes and adverse event data. Full selection criteria are detailed in Supplementary material section 1.

### Data extraction

Titles and abstracts were collated and screened using the Rayyan software^1^. Screening was done independently by two authors (TF and NS), with any discrepancies reviewed by a third party (NAJR). Studies that were identified as meeting the inclusion criteria underwent full paper review which was conducted by two authors (TF and NS), with issues resolved through discussion and consensus with a senior author (NAJR) who made the final decision. A bespoke data collection tool was designed to ensure complete capture of all primary and secondary outcome data points (available on request). Demographic, tumor characteristics, therapeutic and outcome data were collected independently by two authors (TF and NS) and crosschecked (NAJR). The key outcomes are detailed in Supplementary Table S2. In studies in which there were multiple treatment arms, data was only extracted from the relevant arm in which an ICPI has been directly compared to a standard therapy or placebo.

### Assessment of bias

Risk of bias was assessed using the International Cochrane Collaborations guidelines. This tool uses a 3-point scale to assess the following: selection, performance, detection, attrition, and reporting (19). Using scores from each of these 6 domains, an overall risk of bias for each trial was calculated. Definition of overall bias was calculated: ‘low’ if 4 or more domains were scored as low; ‘high’ if 3 or more domains were scored as high; ‘medium’ if neither of the above.

### PD-L1 status

Studies were grouped into two groups; namely those in which the majority of tumors demonstrated high PD-L1 expression and those in which minority of tumors demonstrated high PD-L1 expression. The definition of high PD-L1 expression was taken from the authors’ definition. A majority was defined as >50% of the study population treated with ICPIs had tumors with high PD-L1 expression. In addition, the effect of significant PD-L1 expression (again as defined by the authors) was explored in different subgroup analysis.

### Statistical analysis

*A Priori* power analyses were conducted for Random-Effects (RE) models with a conservative OR of 0.85 and between-study heterogeneity gradings at “low,” “medium,” and “high” with study size n-1 the predicted dataset.

Where present in eligible clinical trials, hazard ratios (HR), risk ratios (RR), standard error (SE) and 95% confidence intervals (CI) were extracted or calculated from source data. Means were standardized across any heterogenous scale limits. See Supplementary material for more detail.

All analysis was performed using R version 4.1.0^2^ with the following libraries: tidyverse, *meta* version 5.0, metafor, and dmetar (20–22).

## Results

### Search results

**Figure 1:**
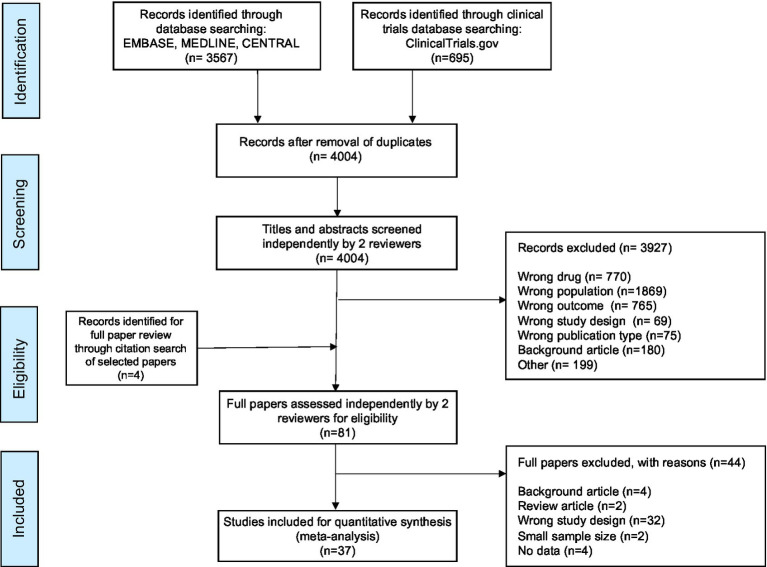
Prisma flow diagram.

Our search of the medical databases yielded 3,567 articles. In addition, searching the trials registry resulted in 695 additional studies. Therefore, after the removal of duplicates (*n* = 258), 4,004 studies underwent initial abstract screening. Four further studies were identified through citation searching. In total 81 papers underwent full manuscript review of which 44 were excluded (see Supplementary Table S4). Therefore 37 studies (23–59) were included in this systematic review and meta-analysis. This process is summarized in Figure 1.

### Study characteristics

In total 46,510 participants were included in the meta-analysis. Four studies (31, 53, 54, 56) were found to have no risk of bias (see Supplementary Figure S1). Included studies were conducted in North America (*n* = 25), China (*n* = 5), Europe (*n* = 4), and Japan (*n* = 3). These studied lung (*n* = 18), gastro-esophageal junction (*n* = 6), head and neck (*n* = 3), breast (*n* = 3), uroepithelial (*n* = 2), ovarian (*n* = 2), liver (*n* = 1), mesothelioma (*n* = 1), and renal cell (*n* = 1) cancers. Studies were either in the primary (*n* = 15) or relapsed (*n* = 22) setting. The majority (*n* = 27) reported the number of tumors with PD1/PD-L1 expression. A range of ICPIs were used: Pembrolizumab (*n* = 16), Nivolumab (*n* = 11), Atezolizumab (*n* = 6) or Avelumab (*n* = 3) Durvalumab (*n* = 1). Most controls were non-ICPI agents, however five studies had a sole placebo control arm. These data are summarized in Table 1. Results detailing analysis power, small study effects, and influential studies can be found in the Supplementary material section 1.4.

**Table 1:** Included studies characteristics.

Author	Study year	Country	Type of cancer	Number of participants	Intervention	Control	Randomization ratio	Primary or relapsed disease	Number of patients with PD1/PDL1 tumors
Gandhi et al.	2018	United States	NSCLC	616	Pembrolizumab	Placebo	2:1	Primary	388
Borghaei et al.	2015	USA	NSCLC	792	Nivolumab	Docetaxel	1:1	Relapsed	246
Zhou et al.	2017	China	NSCLC	425	Pembrolizumab	Docetaxel	1:1	Relapsed	227
Mok et al.	2019	USA	NSCLC	1,274	Pembrolizumab	Carboplatin & Paclitaxel OR Pemetrexed	1:1	Primary	1,274
Reck et al.	2016	China	NSCLC	305	Pembrolizumab	Carboplatin and Paclitaxel OR Gemcitabine and Cisplatin OR Gemcitabine & Carboplatin	1:1	Primary	205
Paz-Ares et al.	2018	Spain	NSCLC	559	Pembrolizumab	Placebo and Paclitaxel OR Nab-paclitaxel	1:1	Primary	353
Vokes et al.	2018	USA	NSCLC	352	Nivolumab	Docetaxel	1:1	Relapsed	42
Barlesi et al.	2018	Germany	NSCLC	792	Avelumab	Docetaxel	1:1	Primary	529
Rittmeyer et al.	2017	USA	NSCLC	1,255	Atezolizumab	Docetaxel	1:1	Primary	463
Carbone et al.	2017	USA	NSCLC	1,325	Nivolumab	Gemcitabine and Paclitaxel OR Pemetrexed	1:1	Relapsed	423
Wu et al.	2019	China	NSCLC	639	Nivolumab	Docetaxel	2:1	Relapsed	252
Pujade-Lauarine et al.	2021	USA	Ovarian	556	Avelumab OR Avelumab and Pegylated Liposomal Doxorubicin	Pegylated Liposomal Doxorubicin	1:1	Relapsed	288
Emens et al.	2021	USA	Breast	902	Atezolizumab and Nab-Paclitaxel	Nab-Paclitaxel and Placebo	1:1	Primary	369
Owonikoko et al.	2021	USA	SCLC	555	Nivolumab	Placebo	1:1	Primary	Unknown
Miles et al.	2021	USA	Breast	651	Atezolizumab and Paclitaxel	Paclitaxel and Placebo	2:1	Primary	292
Spigel et al.	2021	USA	SCLC	803	Nivolumab	Topotecan OR Amrubicin	1:1	Relapsed	Unknown
Shitara et al.	2020	USA	Gastric/GOJ	763	Pembrolizumab OR Pembrolizumab and Cisplatin OR 5-Fluorouracil OR Capecitabine	Placebo and Cisplatin OR 5-Fluorouracil OR Capecitabine	1:1:1	Primary	763
Shitara et al.	2018	USA	Gastric/GOJ	570	Pembrolizumab	Paclitaxel	1:1	Relapsed	380
Kojima et al.	2020	Japan	Oesophageal/GOJ	628	Pembrolizumab	Paclitaxel, Docetaxel, Irinotecan	1:1	Relapsed	222
Cohen et al.	2019	USA	HNSCC	495	Pembrolizumab	Methotrexate OR Docetaxel OR Cetuximab	1:1	Relapsed	295
Burtness et al.	2019	USA	HNSCC	882	Pembrolizumab OR Pembrolizumab and Cisplatin OR carboplatin and 5-Fluorouracil	EXTREME regimen	1:1:1	Primary	754
Ferris et al.	2016	USA	HNSCC	502	Nivolumab	Methotrexate OR docetaxel OR cetuximab	2:1	Relapsed	Unknown
Kang et al.	2017	Japan	Gastric/GOJ	493	Nivolumab	Placebo	2:1	Relapsed	Unknown
Bang et al.	2018	USA	GOJ	371	Avelumab and BSC	Irinotecan OR Paclitaxel & BSC	1:1	Relapsed	Unknown
Kim et al.	2019	China	Oesophageal/GOJ	123	Pembrolizumab	Paclitaxel OR Docetaxel OR Irinotecan	1:1	Relapsed	54
Fradet et al.	2019	Canada	Urothelial	542	Pembrolizumab	Paclitaxel OR Docetaxel OR Vinflunine	1:1	Relapsed	230
Winer et al.	2021	USA	Triple Negative Breast	622	Pembrolizumab	Capecitabine OR Eribulin, Gemcitabine OR Vinorelbine	1:1	Relapsed	605
Rudin et al.	2020	USA	SCLC	453	Pembrolizumab & Etoposide	Placebo & Etoposide	1:1	Primary	Unknown
Finn et al.	2020	USA	Hepatocellular	413	Pembrolizumab	Placebo and BSC	2:1	Relapsed	Unknown
Powles et al.	2018	United Kingdom	Urothelial Bladder	931	Atezolizumab	Paclitaxel OR Docetaxel OR Vinflunine	1:1	Relapsed	Unknown
Hamanishi et al.	2021	Japan	Ovarian	316	Nivolumab	Gemcitabine OR Pegylated Liposomal Doxorubicin	1:1	Relapsed	123
Jassem et al.	2021	USA	NSCLC	572	Atezolizumab	Cisplatin OR Carboplatin and Pemetrexed OR Gemcitabine	1:1	Primary	554
Motzer et al.	2015	USA	Renal Cell Carcinoma	1,068	Nivolumab	Everolimus	1:1	Relapsed	181
Horn et al.	2018	USA	SCLC	403	Atezolizumab & Carboplatin & Etoposide	Placebo and Carboplatin and Etoposide	1:1	Primary	Unknown
Antonia et al.	2018	USA	NSCLC	713	Durvulumab	Placebo	2:1	Relapsed	Unknown
Fennell et al.	2021	United Kingdom	Mesothelioma	332	Nivolumab	Placebo	2:1	Relapsed	86
Wu et al.	2021	China	NSCLC	262	Pembrolizumab	Carboplatin and Paclitaxel OR Pemetrexed	1:1	Primary	262

GOJ, gastro-oesophageal junction adenocarcinoma; HNSCC, head and neck squamous cell carcinoma; SCLC, small cell lung cancer; NSCLC, non-small cell lung cancer; EXTREME regimen, cetuximab, cisplatin and/or carboplatin, 5-fluorouracil; BSC, best supportive care.

### Overall survival

#### All cancers

**Figure 2:**
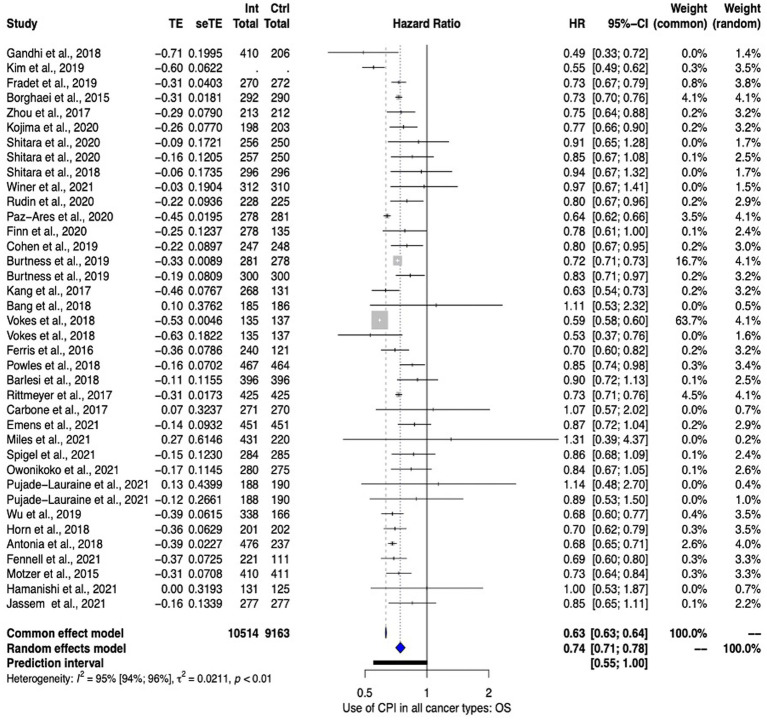
Pan cancer overall survival analysis ICPIs vs Control.

In total, 34 studies informed the pan-cancer overall survival (OS) meta-analysis, with four studies (26, 40, 44, 47) contributing data from more than one arm. Overall, meta-analysis favored the use of ICPIs with a HR of 0.74 (95% CI 0.71–0.78). Heterogeneity was high [*I*^2^ 95% (95% CI 94–96)]. These data are summarized in Figure 2.

#### Cancer site

**Figure 3:**
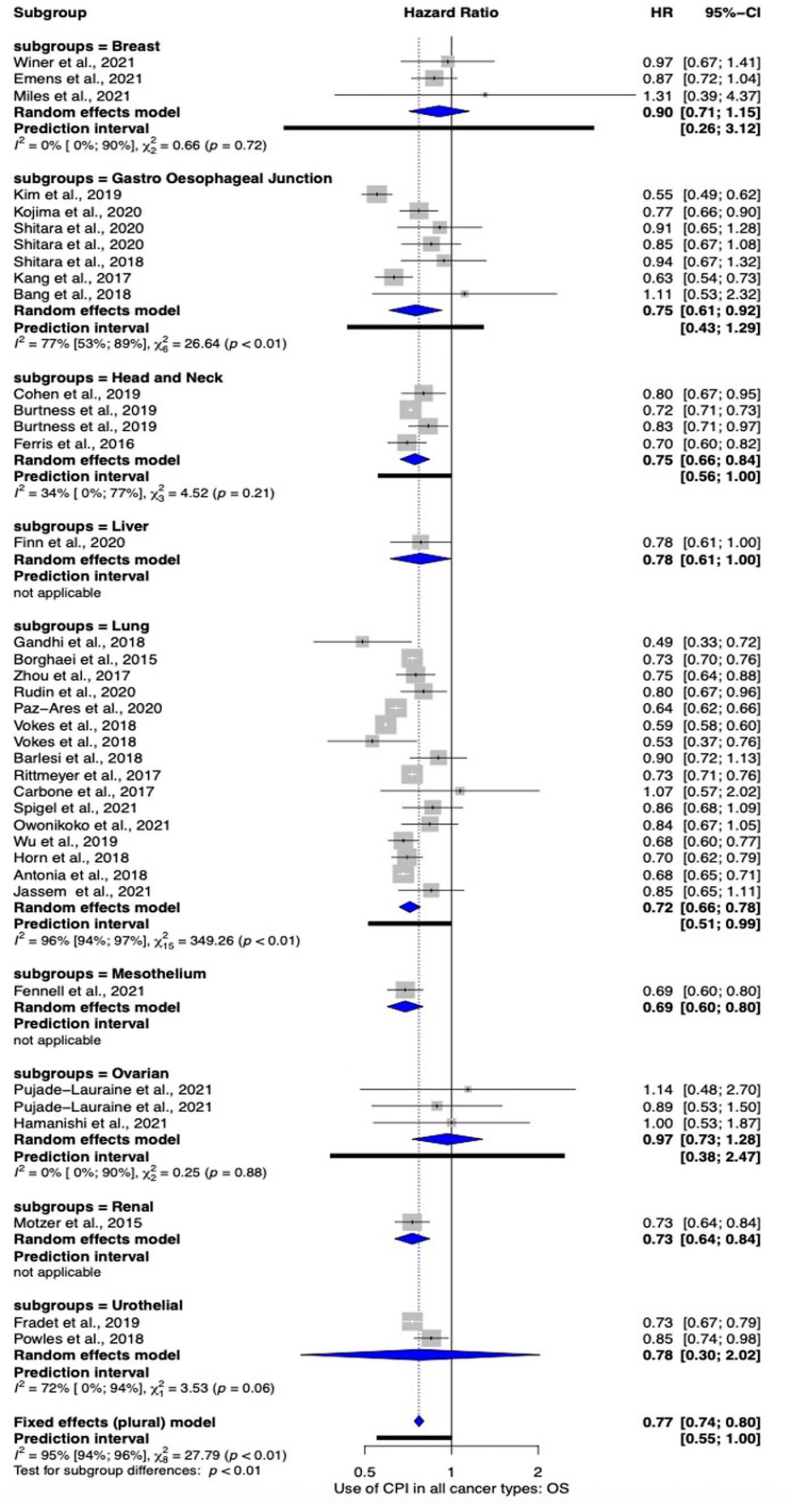
Subgroup meta-analysis comparing the efficacy of ICPI use on OS by cancer site.

Subgroup analysis was performed by cancer site as to explore its effect on OS. The subgroup analysis included studies investigating lung (*n* = 15), gastro-esophageal junction (*n* = 6), breast (*n* = 3), head and neck (*n* = 3), ovarian (*n* = 2), urothelial cancers (*n* = 2), liver (*n* = 1), renal (*n* = 1), and mesothelioma (*n* = 1). Overall, all cancer sites favored the use of ICPIs [HR 0.78 (95% CI 0.74–0.81)]. However, only lung, head and neck, liver, mesothelioma, renal and gastro-esophageal junction cancers demonstrated a significant benefit in their subgroup analysis. Mesothelioma demonstrated the most significant effect [HR 0.69 (95% 0.60–0.80)] however this was based on one study. Where meta-analysis was possible, lung cancers showed the most benefit [HR 0.72 (95% CI 0.66–0.78)] followed by head and neck cancers [HR 0.75 (95% CI 0.66–0.84)] and gastro-esophageal junction cancers [HR 0.75 (95% CI 0.61 to 0.92)]. These data are summarized in Figure 3.

Looking to lung cancer in more detail, a subgroup analysis was performed comparing the effect of ICPIs in both small cell and non-small disease. This was due to the higher levels of tumor mutational burden (TMB) in small cell cancers versus non-small cancers (11). Both histotypes demonstrated a significant benefit favoring the use of ICPIs [HR 0.73 (CI 95% 0.68–0.78)] however the effect was more pronounced in non-small cell cancers vs. small cell cancers (HR 0.69 vs. 0.77 respectively). These data are summarized in Supplementary Figure S2.

### Disease specific characteristics

Immune checkpoint inhibitors seem to be efficacious at both primary presentation and recurrence {[HR 0.73 (95% CI 0.68 to 0.77)] vs. [HR 0.79 (95% CI 0.72 to 0.87)] respectively}. In advanced and metastatic cancers, the use of ICPIs led to an improved OS [HR 0.73 (95% CI 0.68–0.79)]. This was less pronounced in the recurrence setting [HR 0.80 (95% CI 0.72–0.89)] however fewer studies informed this sub meta-analysis when compared to the primary presentation. These data are presented in Supplementary Figure S3. See 1.4 in Supplementary material.

### Treatment characteristics

**Figure 4:**
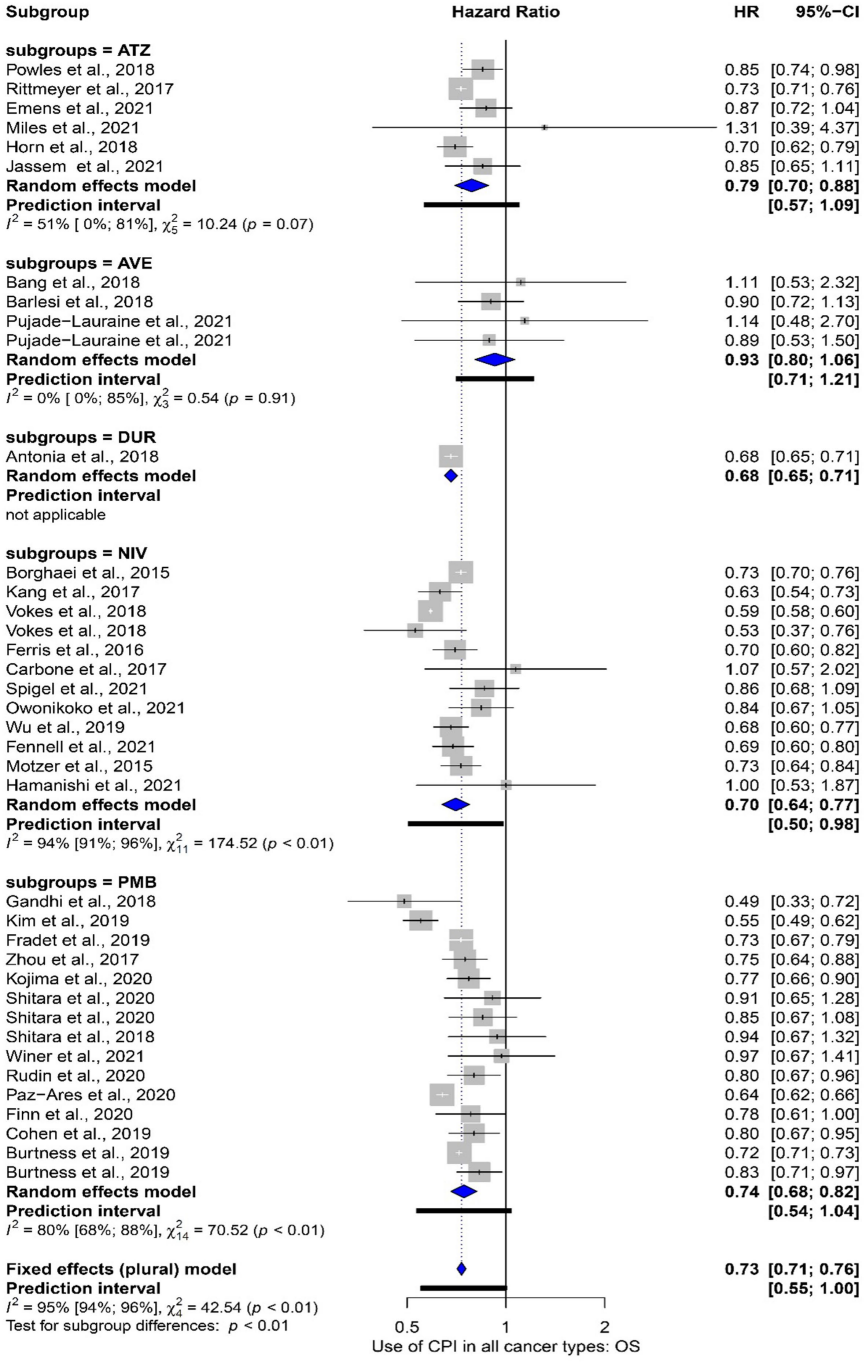
Subgroup meta-analysis comparting the overall survival grouped by studies that used different ICPI agents. Abbreviations: ATZ: Atezolizumab; AVE: Avelumab; NIV: Nivolumab; PMB: Pembrolizumab.

Subgroup analysis was performed comparing the impact on OS subdivided by the specific ICPI used. Where meta-analysis was performed, Nivolumab lead to the greatest impact [HR 0.70 (95% CI 0.64 to 0.77)] with Avelumab failing to reach significance [HR 0.93 (95% CI 0.80 to 1.06)]. However, overall heterogenicity was high (*I*^2^ = 95%). These data are presented in Figure 4. Furthermore, we explored the impact on OS in studies whereby a single ICPI was used vs. studies in which more than one ICPI was used. The impact was similar in both scenarios [single agent HR: 0.68 (95% CI 0.62–0.75) vs. multiple agent HR: 0.68 (95% CI 0.57–0.81)]. These data are shown in Supplementary Figure S6.

### Study characteristics

The impact on OS was compared in studies deemed to be low bias vs. those studies deemed to have a high bias. There was limited impact on OS [low bias HR 0.72 (95% CI 0.66–0.78) vs. high bias HR 0.76 (95% CI 0.71–0.81)]. However, the low bias subgroup had a lower degree of heterogeneity (*I*^2^ 77% vs. 94%). These data are presented in Supplementary Figure S7. Furthermore, we explored the impact of a placebo arm. When compared to a placebo or other treatment, ICPI performed well leading to significant improvement in OS HR 0.74 (95% CI 0.71–0.78). This was, as expected, more pronounced in the placebo arm with an of HR 0.67 (95% CI 0.56–0.82) vs. HR 0.75 (95% CI 0.71–0.80). Only a limited number of studies that included a placebo arm (*n* = 5); these data are summarized in Supplementary Figure S8.

### Progression free survival

#### All cancers

**Figure 5:**
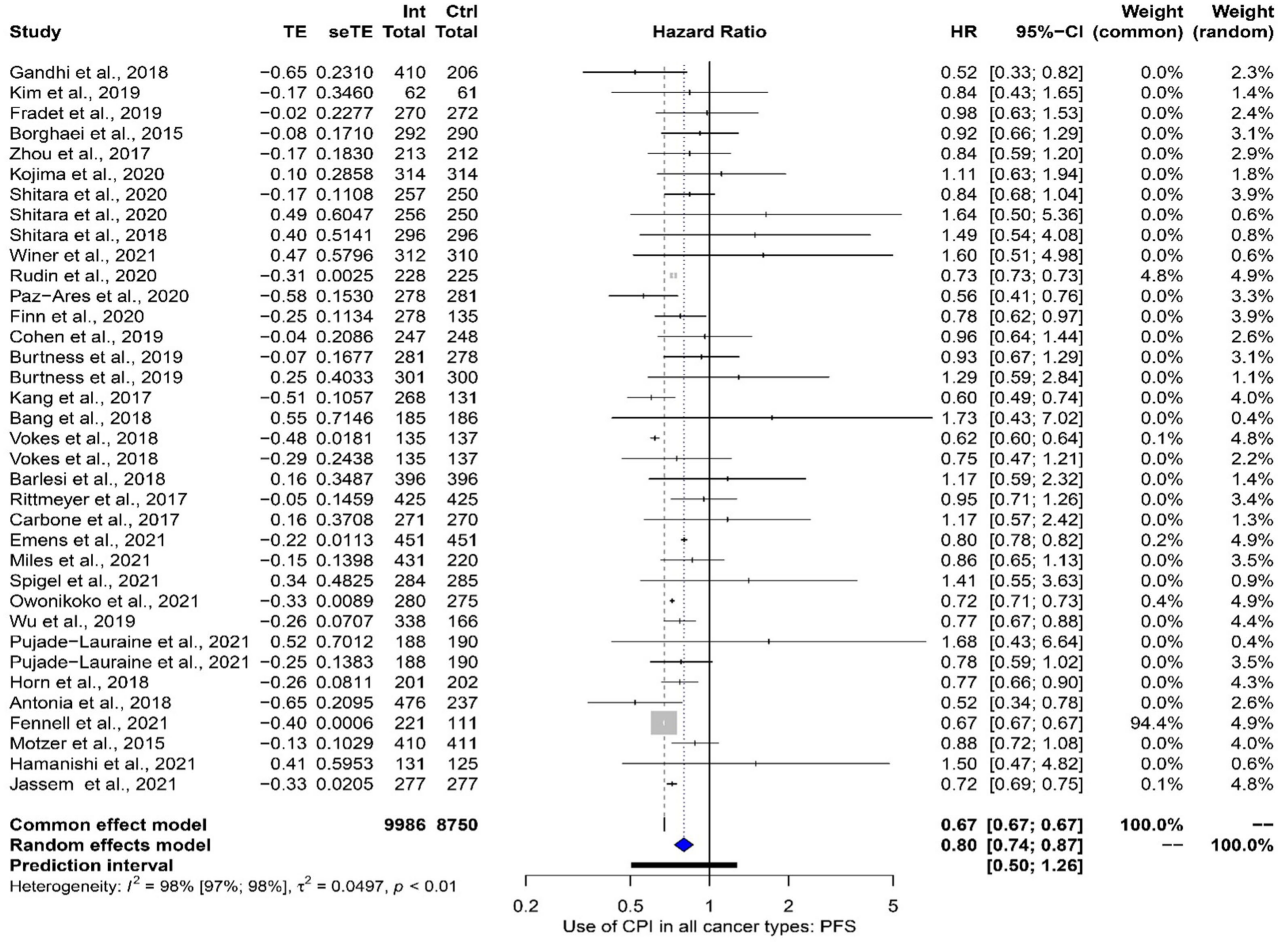
A meta-analysis exploring the impact of ICPIs on progression free survival across all cancer sites.

In total 32 studies informed the pan-cancer progression free survival (PFS) analysis. Once more, four studies contributed data from more than one study arm (26, 40, 44, 47). The use of ICPIs did improve PFS across all cancer sites [HR 0.80 (95% CI 0.74–0.87)]. Heterogeneity across studies was high (*I*^2^ 98%). These data are presented in Figure 5.

#### Cancer site

Studies examining the impact of ICPIs on the PFS of the lung (*n* = 15), gastro-esophageal junction (*n* = 6), breast (*n* = 3), head and neck (*n* = 2), ovarian (*n* = 2), liver (*n* = 1), renal (*n* = 1), mesothelium (*n* = 1), and urothelial (*n* = 1) cancers underwent subgroup analysis to explore the bearing of cancer site on the impact of ICPIs on PFS. Of those in which a meta-analysis was possible, only lung cancers showed a significant improvement in PFS. Looking at lung cancer in more detail, non-small cell studies (*n* = 11) reported a significant improvement [HR 0.73 (95% CI 0.63–0.85)] in PFS with ICPI use. Studies reporting small cell lung cancers (*n* = 4) also reached significance [HR 0.76 (95% CI 0.59–0.97)]. These data are shown in Supplementary Figures S9, S10.

#### Disease specific characteristics

Progression free survival was significantly improved using ICPIs in the primary presentation setting [HR 0.78 (95% CI 0.71–0.86)] however, although there was a trend to improvement in the recurrence setting it did not reach significance [HR 0.85 (95% CI 0.71–1.02)]. These data are outlined in Supplementary Figure S11. PFS was improved with the use of ICPIs in both studies reporting participants who had and had not responded to primary routine treatment {[HR 0.78 (95% CI 0.67–0.89)] and [HR 0.81 (95% CI 0.73–0.91)] respectively – see Supplementary Figure S12}. As seen in OS, the degree of PD-L1 expression did not greatly impact on PFS {[majority PD-L1 HR 0.83 (95% CI 0.71–0.97) vs. minority PD-L1 HR 0.78 (95% CI 0.71 to 0.87)] – see Figure S13}. This is explored in more detail in Supplementary material section 1.5.

#### Treatment characteristics

Regarding specific ICPI use, the PFS mirrored OS with Nivolumab demonstrating the greatest impact [HR 0.75 (95% CI 0.65–0.87)] and Avelumab failing to reach significance [HR 0.97 (95% CI 0.57–1.67)] as shown in Supplementary Figure S14. PFS only showed a significant improvement in the meta-analysis single agent studies [HR 0.78 (95% CI 0.71–0.86)]. In combination ICPI therapy there was a trend toward improved PFS, however this failed to reach significance [HR 0.86 (95% 0.70–1.06)]. Single agent studies had a higher heterogenicity (*I*^2^ 98%) compared with studies with that explored multiple agent use (*I*^2^ 45%). These data are shown in Supplementary Figure S15.

#### Study characteristics

Subgroup analysis demonstrated that overall PFS was improved by ICPI use in studies judged to be high and low bias [HR 0.80 (95% CI 0.74–0.86)]. However, low bias studies demonstrated a greater effect (HR 0.73 vs. 0.85) but with a higher heterogenicity (*I*^2^ 99% vs. 77%). These data are summarized in Supplementary Figure S16. Studies that compared ICPIs with placebo found a significant improvement in PFS [HR 0.65 (95% CI 0.56–0.76)]. In the meta-analysis of ICPIs compared to standard treatment, a significant but less pronounced improvement was seen [HR 0.83 (95% CI 0.77–0.90)]. Five studies made up the placebo meta-analysis (32, 33, 37, 47, 54). These data are shown in Supplementary Figure S17.

#### Side effect profile

**Figure 6:**
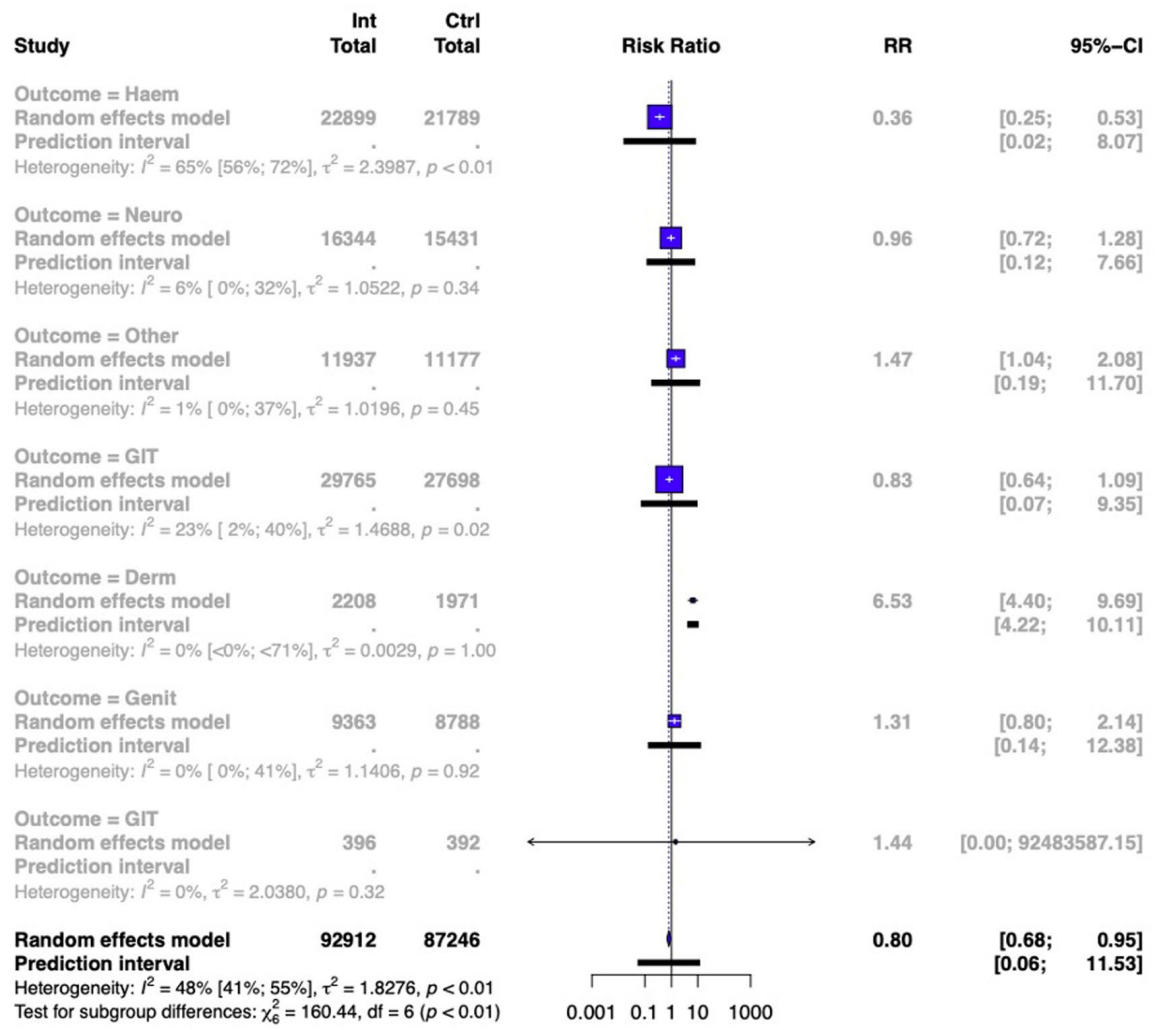
Summary forest plot showing the meta-analysis of reported side effects when comparing ICPI use vs standard treatment/placebo in all cancers.

Overall, a significantly improved side effect profile with ICPI use however was demonstrated [RR 0.80 (95% CI 0.68–0.95)]. A summary meta-analysis is shown in Figure 6 and described in more detail in Supplementary material section 1.4. It should be noted the former side effects were only reported by a few studies.

#### Quality of life outcomes

Too few studies reported quality of life outcomes for a meta-analysis or an informative narrative analysis. Only Pujade-Lauraine et al. made specific reference to quality-of-life outcomes, stating that “treatment related symptom burden was generally similar across all groups” (40).

## Discussion

Immune checkpoint inhibitors are considered tumor-agnostic therapies (60). However, to date, their efficacy has only been studied in RCTs in single tumor sites with limited attempts to describe their therapeutic effect across multiple cancer sites. Furthermore, PD-L1 has been accepted as the biomarker of choice for directing IPCI therapy without any pan-cancer level analysis. To the authors’ knowledge, here we present the most comprehensive review of ICPI use as a tumor-agnostic treatment. We found the use of ICPIs lead to improved survival outcomes across numerous tumor sites with limited toxicity. This was most pronounced in lung, head and neck and gastro-esophageal junction cancers. Our data would suggest the use of ICPI is most effective in the primary treatment setting however it was still beneficial in recurrent disease. Furthermore, ICPI are of benefit in those who have, and have not responded to first line treatment. These findings do not seem to be influenced by the number of ICPIs or the specific ICPI that is used. Finally, PD-L1 as a biomarker of ICPI treatment efficacy would seem problematic.

The variation in ICPI efficacy by cancer site that is seen within our data can be explained mechanistically. ICPIs prevents tumor escape through the PD1/PD-L1 axis which is commonly utilized by cancers with a high mutational burden, as these malignancies express high levels of neoantigens that stimulate a cytotoxic immune response (7, 61) We observed an improved ICPI efficacy in lung, head and neck and gastro-esophageal junction cancers which all have a relatively high mutational burden (62). The improved immunotherapeutic effect in non-small cell lung cancer is well documented (63) and was seen in our data; these cancers also have a hypermutated phenotype (62). Furthermore, we noted primary metastatic cancers had a better response ICPIs; again, metastatic disease is known to have a high mutational burden (64).

Not all studies reported a favorable outcome. Bang et al. (23), Carbone et al. (27), and Miles et al. (35) reported no significant improvement in survival. These outliers can be explained by methodological issues within the studies. Bang et al. stained for PD-L1 with several different antibodies. Of note, subgroup analysis found those cases in which 22C3 antibodies were used for defining PD-L1 expression had better survival outcomes (65); this could speak to a methodological issue rather than a true negative result. Carbone et al. suffered from significant treatment cross over with over 60% of their control group receiving an ICPI. Mature survival data from Miles et al. did find that ICPI use was associated with an improved survival (66).

The use of biomarkers to direct treatment is a tenant of personalized therapy. We explored the effect of using PD-L1 as a biomarker for ICPI use. In studies in which the authors had preselected their treatment cohort based on positive PD-L1 expression, an improved OS HR was noted. However, in head-to-head comparison between studies in which most cancers had a high PD-L1 expression vs. those with a minority where PD-L1 positive, our analysis would indicate that using PD-L1 as a ICPI treatment biomarker was of deleterious effect, although this did not reach significance. Given these data are not consistent it may suggest PD-L1 may not be as reliable a predictor of ICPI effectiveness as commonly held. The immunohistochemistry for PD-L1 is known to be difficult and often open to high levels of result inconsistency (67–69). Issues with fixation, antibody binding, clonal expression, and interpretation are established issues (70, 71) The included studies within this meta-analysis used a range of immunohistochemical platforms, antibodies, scoring methods, thresholds and number of pathologists involved in forming a consensus opinion. This therefore reflects the real word situation in which PD-L1 testing and scoring is heterogeneous (72). The authors therefore recommend that PD-L1 is not preferentially used as to direct ICPI treatment as it does not seem to improve survival outcomes. Other biomarkers, with higher levels of test consistency, such as TMB and or MMRd either via immunohistochemistry or MSI testing should be explored as potential biomarkers. Meta-analysis exploring the utility of TMB as a biomarker of ICPI treatment concluded it led to a significant improvement in survival outcomes, however this analysis relied on retrospective trial data (73). Tumor mutational analysis has been used to direct ICPI treatment in trials and has been shown to predict significantly improved survival outcomes (73). We were unable to do this in this meta-analysis due to a lack of study data; only one RCT used tumor mutational burden prospectively in our analysis (39).

To put our work in context, Sun et al. published a systematic review and meta-analysis on ICPI use in advanced and metastatic cancers including 35 studies in adult and non-adult populations (13). They too found potential issues with PD-L1 as a biomarker for directing ICPI use, however they were unable to draw clear conclusions do to methodological issues. Studies included in this analysis included trials with ICPIs and other biological treatments which makes conclusions on ICPI effect difficult. Subgroup analyses within this work relied on small numbers. In addition, this meta-analysis included a large proportion of melanomas (8%); these cancers are known to demonstrate consistently high PD-L1 expression and therefore could impact on the conclusions of PD-L1 as a biomarker of ICPI efficacy in other cancer sites (74). Overall response rate was included in their analysis, and as noted by the authors, such a measure is thought unhelpful in immunotherapy as cancers often demonstrate a pseudo-progression on ICPI as they increase in size secondary to immune infiltrate (75). Finally, the inclusion of phase II trials is problematic as these studies tend to be underpowered to explore survival outcomes and present immature data which add to the heterogeneity of analysis and make summary statistics more problematic (76). Therefore, our work does add to the current knowledgebase given it applies a more robust inclusion/exclusion criteria and more mature survival data.

A strength of our work is it followed PRISMA guidance throughout (17). Our thorough search and screening lead to the inclusion of 37 phase III randomized control trials for our meta-analysis. This has enabled a comprehensive summary of the current evidence and enabled robust conclusions as to the clinical efficacy of ICPIs across numerous tumor sites and populations. Furthermore, we limited subgroup analysis to remain within our published protocol. These subgroups were comprised of large cohorts of study subjects giving reliability to the analysis. In addition, throughout the meta-analysis, multiple sensitivity studies were conducted to ensure the robustness of results.

Our work is not without its limitations. We used study level data as our attempts to contact authors to collect individual level data were unsuccessful. Furthermore, most studies included in this analysis were not free from methodological bias. We recognize the studies included in this meta-analysis are heterogeneous; the effects of this were mitigated by the use random effects modeling. However, meaningful comparisons in heterogeneous data can be difficult. Of note, because of the available studies, there is a predominance of lung cancers, the use of Pembrolizumab and North American populations which could impact on the generalizability of this meta-analysis and its conclusions. In addition, we were unable to include studies without an English translation which introduces selection bias within our study. In addition, there are several studies that have or are due to publish exploring ICPI use in novel cancer sites that had not published at the time of our search; these are therefore not included in this analysis. These factors, individually and combined, decrease the reliability of this analysis. However, even with these limitations, our work remains the most comprehensive meta-analysis of ICPI use in solid cancers published to date.

We did not include melanomas in this study. This was to reduce the confounding effect of their inclusion on this analysis; melanomas have an excellent response to ICPIs which is well established (18). In addition, the staining for PD-L1 has been extensively explored in this cancer meaning that PD-L1 interpretation benefits from established protocols (77, 78). PD-L1 is often expressed at high levels in melanoma which also aids in the interpretation of this biomarker in a way that is not so in other cancer sites (77–79). Therefore, through the exclusion of melanomas, our meta-analysis can better explore the impact of ICPIs in non-melanoma solid cancers which we believe have distinct clinical characteristics. However, we recognize it does mean the findings of this study cannot be applied to melanomas.

## Conclusion

In conclusion, herein we present the most comprehensive review of ICPIs as a tumor agnostic therapy. These data confirm that their use improves survival outcomes across a range of tumor sites and are well tolerated. This benefit is seen regardless of specific ICPI used, whether in primary or recurrent disease and where there had and had not been a good response to primary treatment. Of note, the use of PD-L1 immunohistochemistry to direct ICPI use would seem potentially problematic and other biomarkers such as TMB or mismatch repair status should be explored in more depth. In addition, trials should be conducted in cancers that have been so far under investigated.

## Data availability statement

The original contributions presented in the study are included in the article/Supplementary material, further inquiries can be directed to the corresponding authors.

## Author contributions

TF, NS, TW and NR contributed to the data acquisition, data interpretation and data analysis. NR drafted the manuscript. All authors contributed to the literature search, study concept and design, and critical revision to the manuscript.

## Funding

EC was supported by the NIHR Manchester Biomedical Research Centre (IS-BRC-1215-2007) and an NIHR advanced fellowship (NIHR300650). DE is an NIHR Senior Investigator (NF-SI-0513-10076).

## Conflict of interest

The authors declare that the research was conducted in the absence of any commercial or financial relationships that could be construed as a potential conflict of interest.

## Publisher’s note

All claims expressed in this article are solely those of the authors and do not necessarily represent those of their affiliated organizations, or those of the publisher, the editors and the reviewers. Any product that may be evaluated in this article, or claim that may be made by its manufacturer, is not guaranteed or endorsed by the publisher.

## Footnotes

^1^ https://rayyan.qcri.org/

^2^ www.R-project.org/

## Abbreviations

CI: confidence Interval
FDA: Federal Drug Administration
HMB: high mutational burden
HR: hazard ratio
ICPI: immune checkpoint inhibitors
MSI: microsatellite instability
OS: overall survival
PD-1: programmed death 1
PD-L1: programmed death-ligand 1
PFS: progression free survival
PRISMA: preferred reporting items for systematic reviews and meta-analyses
RCT: randomized control trial
RE: random effects
RR: risk ratio
SE: standard error

## References

[1] GBD 2017 Causes of Death Collaborators. Global, regional, And national age-sex-specific mortality for 282 causes of death in 195 countries and territories, 1980-2017: a systematic analysis for the global burden of disease study 2017. Lancet. (2018) 392:1736–88. doi: 10.1016/s0140-6736(18)32203-7, PMID: 30496103 PMC6227606

[2] Nagai H Kim YH. Cancer prevention from the perspective of global cancer burden patterns. J Thorac Dis. (2017) 9:448–51. doi: 10.21037/jtd.2017.02.75 28449441 PMC5394024

[3] Gerber DE. Targeted therapies: a new generation of cancer treatments. Am Fam Physician. (2008) 77:311–9. Available at: https://pubmed.ncbi.nlm.nih.gov/18297955/ PMID: 18297955

[4] Pardoll DM. The blockade of immune checkpoints in cancer immunotherapy. Nat Rev Cancer. (2012) 12:252–64. doi: 10.1038/nrc3239, PMID: 22437870 PMC4856023

[5] le DT Uram JN Wang H Bartlett BR Kemberling H Eyring AD. PD-1 blockade in tumors with mismatch-repair deficiency. N Engl J Med. (2015) 372:2509–20. doi: 10.1056/nejmoa1500596, PMID: 26028255 PMC4481136

[6] Keir ME Butte MJ Freeman GJ Sharpe AH. PD-1 and its ligands in tolerance and immunity. Annu Rev Immunol. (2008) 26:677–704. doi: 10.1146/annurev.immunol.26.021607.090331 18173375 PMC10637733

[7] Ramchander NC Ryan NAJ Walker TDJ Harries L Bolton J Bosse T. Distinct immunological landscapes characterize inherited and sporadic mismatch repair deficient endometrial Cancer. Front Immunol. (2020) 10:3023. doi: 10.3389/fimmu.2019.03023, PMID: 31998307 PMC6970202

[8] Nair VS Elkord E. Immune checkpoint inhibitors in cancer therapy: a focus on T-regulatory cells. Immunol Cell Biol. (2017) 96:21–33. doi: 10.1111/imcb.1003 29359507

[9] Thallinger C Füreder T Preusser M Heller G Müllauer L Höller C. Review of cancer treatment with immune checkpoint inhibitors. Wien Klin Wochenschr. (2018) 130:85–91. doi: 10.1007/s00508-017-1285-9, PMID: 29098404 PMC5816095

[10] Marcus L Lemery SJ Keegan P Pazdur R. FDA approval summary: Pembrolizumab for the treatment of microsatellite instability-high solid tumors. Clin Cancer Res Official J Am Assoc Cancer Res. (2019) 25:3753–8. doi: 10.1158/1078-0432.ccr-18-4070, PMID: 30787022

[11] Sha D Jin Z Budczies J Kluck K Stenzinger A Sinicrope FA. Tumor mutational burden as a predictive biomarker in solid tumors. Cancer Discov. (2020) 10:1808–25. doi: 10.1158/2159-8290.cd-20-0522 33139244 PMC7710563

[12] Twomey JD Zhang B. Cancer immunotherapy update: FDA-approved checkpoint inhibitors and companion diagnostics. AAPS J. (2021) 23:39. doi: 10.1208/s12248-021-00574-0, PMID: 33677681 PMC7937597

[13] Sun L Zhang L Yu J Zhang Y Pang X Ma C. Clinical efficacy and safety of anti-PD-1/PD-L1 inhibitors for the treatment of advanced or metastatic cancer: a systematic review and meta-analysis. Sci Rep. (2020) 10:2083. doi: 10.1038/s41598-020-58674-4 32034198 PMC7005709

[14] Robert C. A decade of immune-checkpoint inhibitors in cancer therapy. Nat Commun. (2020) 11:3801. doi: 10.1038/s41467-020-17670-y, PMID: 32732879 PMC7393098

[15] Larkin J Chiarion-Sileni V Gonzalez R Grob JJ Rutkowski P Lao CD. Five-year survival with combined Nivolumab and Ipilimumab in advanced melanoma. New Engl J Med. (2019) 381:1535–46. doi: 10.1056/nejmoa1910836, PMID: 31562797

[16] Haslam A Prasad V. Estimation of the percentage of US patients with Cancer who are eligible for and respond to checkpoint inhibitor immunotherapy drugs. JAMA Netw Open. (2019) 2:e192535. doi: 10.1001/jamanetworkopen.2019.2535, PMID: 31050774 PMC6503493

[17] Page MJ McKenzie JE Bossuyt PM Boutron I Hoffmann TC Mulrow CD. The PRISMA 2020 statement: an updated guideline for reporting systematic reviews. BMJ. (2021) 372:n71. doi: 10.1136/bmj.n71, PMID: 33782057 PMC8005924

[18] Huang AC Zappasodi R. A decade of checkpoint blockade immunotherapy in melanoma: understanding the molecular basis for immune sensitivity and resistance. Nat Immunol. (2022) 23:660–70. doi: 10.1038/s41590-022-01141-1, PMID: 35241833 PMC9106900

[19] Shemilt I Aluko P Graybill E Craig D Henderson C Drummond M. Cochrane handbook for systematic reviews of interventions. The Cochrane Collaboration and John Wiley & Sons Ltd. (2019) 507–523

[20] Balduzzi S Rücker G Schwarzer G. How to perform a meta-analysis with R: a practical tutorial. Evid Based Ment Heal. (2019) 22:153–60. doi: 10.1136/ebmental-2019-300117, PMID: 31563865 PMC10231495

[21] Wickham H Averick M Bryan J Chang W McGowan L François R. Welcome to the Tidyverse. J Open Source Softw. (2019) 4:1686. doi: 10.21105/joss.01686

[22] Viechtbauer W. Conducting Meta-analyses in R with the metafor package. J Stat Softw. (2010) 36:1–42. doi: 10.18637/jss.v036.i03

[23] Bang YJ Ruiz EY van Cutsem E Lee KW Wyrwicz L Schenker M. Phase III, randomised trial of avelumab versus physician’s choice of chemotherapy as third-line treatment of patients with advanced gastric or gastro-oesophageal junction cancer: primary analysis of JAVELIN gastric 300. Ann Oncol. (2018) 29:2052–60. doi: 10.1093/annonc/mdy264, PMID: 30052729 PMC6225815

[24] Barlesi F Vansteenkiste J Spigel D Ishii H Garassino M de Marinis F. Avelumab versus docetaxel in patients with platinum-treated advanced non-small-cell lung cancer (JAVELIN lung 200): an open-label, randomised, phase 3 study. Lancet Oncol. (2018) 19:1468–79. doi: 10.1016/s1470-2045(18)30673-9 30262187

[25] Borghaei H Paz-Ares L Horn L Spigel DR Steins M Ready NE. Nivolumab versus docetaxel in advanced nonsquamous non–small-cell lung Cancer. New Engl J Med. (2015) 373:1627–39. doi: 10.1056/nejmoa1507643, PMID: 26412456 PMC5705936

[26] Burtness B Harrington KJ Greil R Soulières D Tahara M de Castro G Jr. Pembrolizumab alone or with chemotherapy versus cetuximab with chemotherapy for recurrent or metastatic squamous cell carcinoma of the head and neck (KEYNOTE-048): a randomised, open-label, phase 3 study. Lancet. (2019) 394:1915–28. doi: 10.1016/s0140-6736(19)32591-7 31679945

[27] Carbone DP Reck M Paz-Ares L Creelan B Horn L Steins M. First-line Nivolumab in stage IV or recurrent non-small-cell lung Cancer. New Engl J Medicine. (2017) 376:2415–26. doi: 10.1056/nejmoa1613493, PMID: 28636851 PMC6487310

[28] Cohen EEW Soulières D le Tourneau C Dinis J Licitra L Ahn MJ. Pembrolizumab versus methotrexate, docetaxel, or cetuximab for recurrent or metastatic head-and-neck squamous cell carcinoma (KEYNOTE-040): a randomised, open-label, phase 3 study. Lancet. (2019) 393:156–67. doi: 10.1016/s0140-6736(18)31999-8 30509740

[29] Emens LA Adams S Barrios CH Diéras V Iwata H Loi S. First-line atezolizumab plus nab-paclitaxel for unresectable, locally advanced, or metastatic triple-negative breast cancer: IMpassion130 final overall survival analysis. Ann Oncol. (2021) 32:983–93. doi: 10.1016/j.annonc.2021.05.355 34272041

[30] Ferris RL Blumenschein G Fayette J Guigay J Colevas AD Licitra L. Nivolumab for recurrent squamous-cell carcinoma of the head and neck. N Engl J Med. (2016) 375:1856–67. doi: 10.1056/nejmoa1602252 27718784 PMC5564292

[31] Finn RS Ryoo BY Merle P Kudo M Bouattour M Lim HY. Pembrolizumab As second-line therapy in patients with advanced hepatocellular carcinoma in KEYNOTE-240: a randomized, double-blind, phase III trial. J Clin Oncol Official J Am Soc Clin Oncol. (2019) 38:193–202. doi: 10.1200/jco.19.01307, PMID: 31790344

[32] Gandhi L Rodríguez-Abreu D Gadgeel S Esteban E Felip E de Angelis F. Pembrolizumab plus chemotherapy in metastatic non–small-cell lung Cancer. New Engl J Med. (2018) 378:2078–92. doi: 10.1056/nejmoa1801005 29658856

[33] Kang YK Boku N Satoh T Ryu MH Chao Y Kato K. Nivolumab in patients with advanced gastric or gastro-oesophageal junction cancer refractory to, or intolerant of, at least two previous chemotherapy regimens (ONO-4538-12, ATTRACTION-2): a randomised, double-blind, placebo-controlled, phase 3 trial. Lancet. (2017) 390:2461–71. doi: 10.1016/s0140-6736(17)31827-5 28993052

[34] Kojima T Shah MA Muro K Francois E Adenis A Hsu CH. Randomized phase III KEYNOTE-181 study of Pembrolizumab versus chemotherapy in advanced esophageal Cancer. J Clin Oncol Official J Am Soc Clin Oncol. (2020) 38:4138–48. doi: 10.1200/jco.20.01888, PMID: 33026938

[35] Miles D Gligorov J André F Cameron D Schneeweiss A Barrios C. Primary results from IMpassion131, a double-blind, placebo-controlled, randomised phase III trial of first-line paclitaxel with or without atezolizumab for unresectable locally advanced/metastatic triple-negative breast cancer. Ann Oncol. (2021) 32:994–1004. doi: 10.1016/j.annonc.2021.05.801, PMID: 34219000

[36] Mok TSK Wu YL Kudaba I Kowalski DM Cho BC Turna HZ. Pembrolizumab versus chemotherapy for previously untreated, PD-L1-expressing, locally advanced or metastatic non-small-cell lung cancer (KEYNOTE-042): a randomised, open-label, controlled, phase 3 trial. Lancet Lond Engl. (2019) 393:1819–30. doi: 10.1016/s0140-6736(18)32409-7, PMID: 30955977

[37] Owonikoko TK Park K Govindan R Ready N Reck M Peters S. Nivolumab and Ipilimumab as maintenance therapy in extensive-disease small-cell lung Cancer: CheckMate 451. J Clin Oncol. (2021) 39:1349–59. doi: 10.1200/jco.20.02212 33683919 PMC8078251

[38] Paz-Ares L Luft A Vicente D Tafreshi A Gümüş M Mazières J. Pembrolizumab plus chemotherapy for squamous non–small-cell lung Cancer. New Engl J Med. (2018) 379:2040–51. doi: 10.1056/nejmoa1810865 30280635

[39] Powles T Durán I van der HMS van der Heijden MS Loriot Y Vogelzang NJ. Atezolizumab versus chemotherapy in patients with platinum-treated locally advanced or metastatic urothelial carcinoma (IMvigor211): a multicentre, open-label, phase 3 randomised controlled trial. Lancet. (2018) 391:748–57. doi: 10.1016/s0140-6736(17)33297-x, PMID: 29268948

[40] Pujade-Lauraine E Fujiwara K Ledermann JA Oza AM Kristeleit R Ray-Coquard IL. Avelumab alone or in combination with chemotherapy versus chemotherapy alone in platinum-resistant or platinum-refractory ovarian cancer (JAVELIN ovarian 200): an open-label, three-arm, randomised, phase 3 study. Lancet Oncol. (2021) 22:1034–46. doi: 10.1016/s1470-2045(21)00216-3 34143970

[41] Reck M Rodríguez-Abreu D Robinson AG Hui R Csőszi T Fülöp A. Pembrolizumab versus chemotherapy for PD-L1–positive non–small-cell lung Cancer. N Engl J Med. (2016) 375:1823–33. doi: 10.1056/nejmoa1606774 27718847

[42] Rittmeyer A Barlesi F Waterkamp D Park K Ciardiello F von Pawel J. Atezolizumab versus docetaxel in patients with previously treated non-small-cell lung cancer (OAK): a phase 3, open-label, multicentre randomised controlled trial. Lancet. (2017) 389:255–65. doi: 10.1016/s0140-6736(16)32517-x 27979383 PMC6886121

[43] Rudin CM Awad MM Navarro A Gottfried M Peters S Csőszi T. Pembrolizumab or placebo plus etoposide and platinum as first-line therapy for extensive-stage small-cell lung Cancer: randomized, double-blind, phase III KEYNOTE-604 study. J Clin Oncol. (2020) 38:2369–79. doi: 10.1200/jco.20.00793, PMID: 32468956 PMC7474472

[44] Shitara K van Cutsem E Bang YJ Fuchs C Wyrwicz L Lee KW. Efficacy and safety of Pembrolizumab or Pembrolizumab plus chemotherapy vs chemotherapy alone for patients with first-line, advanced gastric Cancer. JAMA Oncol. (2020) 6:1571–80. doi: 10.1001/jamaoncol.2020.3370 32880601 PMC7489405

[45] Shitara K Özgüroğlu M Bang YJ di Bartolomeo M Mandalà M Ryu MH. Pembrolizumab versus paclitaxel for previously treated, advanced gastric or gastro-oesophageal junction cancer (KEYNOTE-061): a randomised, open-label, controlled, phase 3 trial. Lancet. (2018) 392:123–33. doi: 10.1016/s0140-6736(18)31257-1, PMID: 29880231

[46] Spigel DR Vicente D Ciuleanu TE Gettinger S Peters S Horn L. Second-line nivolumab in relapsed small-cell lung cancer: check mate 331. Ann Oncol Official J European Soc Medical Oncol. (2021) 32:631–41. doi: 10.1016/j.annonc.2021.01.071, PMID: 33539946

[47] Vokes EE Ready N Felip E Horn L Burgio MA Antonia SJ. Nivolumab versus docetaxel in previously treated advanced non-small-cell lung cancer (check mate 017 and check mate 057): 3-year update and outcomes in patients with liver metastases. Ann Oncol. (2018) 29:959–65. doi: 10.1093/annonc/mdy041, PMID: 29408986

[48] Winer EP Lipatov O Im SA Goncalves A Muñoz-Couselo E Lee KS. Pembrolizumab versus investigator-choice chemotherapy for metastatic triple-negative breast cancer (KEYNOTE-119): a randomised, open-label, phase 3 trial. Lancet Oncol. (2021) 22:499–511. doi: 10.1016/s1470-2045(20)30754-3 33676601

[49] Wu YL Lu S Cheng Y Zhou C Wang J Mok T. Nivolumab versus docetaxel in a predominantly Chinese patient population with previously treated advanced NSCLC: CheckMate 078 randomized phase III clinical trial. J Thorac Oncol. (2019) 14:867–75. doi: 10.1016/j.jtho.2019.01.006 30659987

[50] NCT02864394. Study of pembrolizumab versus docetaxel in participants previously treated for non-small cell lung Cancer (MK-3475-033/KEYNOTE-033) (2022). Available at: https://clinicaltrials.gov/ct2/show/NCT02864394 (Accessed December 17, 2021).

[51] NCT03933449. Study of pembrolizumab (MK-3475) versus investigator’s choice of chemotherapy for participants with advanced esophageal/esophagogastric junction carcinoma that progressed after first-line therapy (MK-3475-181/KEYNOTE-181)-China extension study. (2019). Available at: https://clinicaltrials.gov/ct2/show/NCT03933449 (Accessed December 17, 2021)

[52] Fradet Y Bellmunt J Vaughn DJ Lee JL Fong L Vogelzang NJ. Randomized phase III KEYNOTE-045 trial of pembrolizumab versus paclitaxel, docetaxel, or vinflunine in recurrent advanced urothelial cancer: results of > 2 years of follow-up. Ann Oncol. (2019) 30:970–6. doi: 10.1093/annonc/mdz127 31050707 PMC6594457

[53] Antonia SJ Villegas A Daniel D Vicente D Murakami S Hui R. Overall survival with Durvalumab after Chemoradiotherapy in stage III NSCLC. New Engl J Med. (2018) 379:2342–50. doi: 10.1056/nejmoa1809697 30280658

[54] Fennell DA Ewings S Ottensmeier C Califano R Hanna GG Hill K. Nivolumab versus placebo in patients with relapsed malignant mesothelioma (CONFIRM): a multicentre, double-blind, randomised, phase 3 trial. Lancet Oncol. (2021) 22:1530–40. doi: 10.1016/s1470-2045(21)00471-x, PMID: 34656227 PMC8560642

[55] Hamanishi J Takeshima N Katsumata N Ushijima K Kimura T Takeuchi S. Nivolumab versus gemcitabine or Pegylated liposomal doxorubicin for patients with platinum-resistant ovarian Cancer: open-label, randomized trial in Japan (NINJA). J Clin Oncol. (2021) 39:3671–81. doi: 10.1200/jco.21.00334, PMID: 34473544 PMC8601279

[56] Horn L Mansfield AS Szczęsna A Havel L Krzakowski M Hochmair MJ. First-line Atezolizumab plus chemotherapy in extensive-stage small-cell lung Cancer. New Engl J Medicine. (2018) 379:2220–9. doi: 10.1056/nejmoa1809064 30280641

[57] Jassem J de Marinis F Giaccone G Vergnenegre A Barrios CH Morise M. Updated overall survival analysis from IMpower110: Atezolizumab versus platinum-based chemotherapy in treatment-naive programmed death-ligand 1–selected NSCLC. J Thorac Oncol. (2021) 16:1872–82. doi: 10.1016/j.jtho.2021.06.019 34265434

[58] Motzer RJ Rini BI McDermott DF Redman BG Kuzel TM Harrison MR. Nivolumab for metastatic renal cell carcinoma: results of a randomized phase II trial. JCO. (2015) 33:1430–7. doi: 10.1200/jco.2014.59.0703, PMID: 25452452 PMC4806782

[59] Wu YL Zhang L Fan Y Zhou J Zhang L Zhou Q. Randomized clinical trial of pembrolizumab vs chemotherapy for previously untreated Chinese patients with PD-L1-positive locally advanced or metastatic non-small-cell lung cancer: KEYNOTE-042 China study. Int J Cancer. (2020) 148:2313–20. doi: 10.1002/ijc.33399, PMID: 33231285 PMC8048589

[60] Looney AM Nawaz K Webster RM. Tumour-agnostic therapies. Nat Rev Drug Discov. (2020) 19:383–4. doi: 10.1038/d41573-020-00015-1 32494047

[61] Rousseau B Foote MB Maron SB Diplas BH Lu S Argilés G. The Spectrum of benefit from checkpoint blockade in Hypermutated tumors. N Engl J Med. (2021) 384:1168–70. doi: 10.1056/nejmc2031965 33761214 PMC8403269

[62] Alexandrov LB Kim J Haradhvala NJ Huang MN Tian Ng AW Wu Y. The repertoire of mutational signatures in human cancer. Nature. (2020) 578:94–101. doi: 10.1038/s41586-020-1943-3 32025018 PMC7054213

[63] Thai AA Solomon BJ Sequist LV Gainor JF Heist RS. Lung cancer. Lancet. (2021) 398:535–54. doi: 10.1016/s0140-6736(21)00312-3 34273294

[64] Schnidrig D Turajlic S Litchfield K. Tumour mutational burden: primary versus metastatic tissue creates systematic bias. Immuno Oncol Technol. (2019) 4:8–14. doi: 10.1016/j.iotech.2019.11.003, PMID: 35755001 PMC9216665

[65] Moehler M Dvorkin M Boku N Özgüroğlu M Ryu MH Muntean AS. Phase III trial of Avelumab maintenance after first-line induction chemotherapy versus continuation of chemotherapy in patients with gastric cancers: results from JAVELIN gastric 100. J Clin Oncol. (2021) 39:966–77. doi: 10.1200/jco.20.00892, PMID: 33197226 PMC8078426

[66] Emens LA Adams S Barrios CH Dieras VC Iwata H Loi S. IMpassion130: final OS analysis from the pivotal phase III study of atezolizumab + nab-paclitaxel vs placebo + nab-paclitaxel in previously untreated locally advanced or metastatic triple-negative breast cancer. Ann Oncol. (2020) 31:S1148. doi: 10.1016/j.annonc.2020.08.2244

[67] Ruiter EJ de Mulder FJ Koomen BM Speel EJ van den Hout MFCM de Roest RH. Comparison of three PD-L1 immunohistochemical assays in head and neck squamous cell carcinoma (HNSCC). Mod Pathol Official J United States Can Acad Pathology Inc (2020) 34: 1125–1132. doi: 10.1038/s41379-020-0644-7, PMID: 32759978

[68] Hutarew G. PD-L1 testing, fit for routine evaluation? From a pathologist’s point of view. Memo-Mag European Medical Oncol. (2016) 9:201–6. doi: 10.1007/s12254-016-0292-2, PMID: 28058063 PMC5165031

[69] Lou SK Ko HM Kinoshita T MacDonald S Weiss J Czarnecka-Kujawa K. Implementation of PD-L1 22C3 IHC pharmDx<sup>TM</sup> in cell block preparations of lung Cancer: concordance with surgical resections and technical validation of CytoLyt® Prefixation. Acta Cytol. (2020) 64:577–87. doi: 10.1159/000508628 32599583 PMC7677989

[70] Jöhrens K Rüschoff J. The challenge to the pathologist of PD-L1 expression in tumor cells of non-small-cell lung Cancer—an overview. Curr Oncol. (2021) 28:5227–39. doi: 10.3390/curroncol28060437 34940076 PMC8699902

[71] Akhtar M Rashid S Al-Bozom IA. PD-L1 immunostaining: what pathologists need to know. Diagn Pathol. (2021) 16:94. doi: 10.1186/s13000-021-01151-x 34689789 PMC8543866

[72] McLaughlin J Han G Schalper KA Carvajal-Hausdorf D Pelekanou V Rehman J. Quantitative assessment of the heterogeneity of PD-L1 expression in non-small-cell lung Cancer. JAMA Oncol. (2016) 2:46–54. doi: 10.1001/jamaoncol.2015.3638 26562159 PMC4941982

[73] Kim JY Kronbichler A Eisenhut M Hong SH van der Vliet HJ Kang J. Tumor mutational burden and efficacy of immune checkpoint inhibitors: a systematic review and Meta-analysis. Cancers. (2019) 11:1798. doi: 10.3390/cancers11111798 31731749 PMC6895916

[74] Kuryk L Bertinato L Staniszewska M Pancer K Wieczorek M Salmaso S. From conventional therapies to immunotherapy: melanoma treatment in review. Cancers. (2020) 12:3057. doi: 10.3390/cancers12103057, PMID: 33092131 PMC7589099

[75] Ozaki Y Shindoh J Miura Y Nakajima H Oki R Uchiyama M. Serial pseudoprogression of metastatic malignant melanoma in a patient treated with nivolumab: a case report. BMC Cancer. (2017) 17:778. doi: 10.1186/s12885-017-3785-4, PMID: 29162045 PMC5696908

[76] Korn EL Liu PY Lee SJ Chapman JAW Niedzwiecki D Suman VJ. Meta-analysis of phase II cooperative group trials in metastatic stage IV melanoma to determine progression-free and overall survival benchmarks for future phase II trials. J Clin Oncol. (2008) 26:527–34. doi: 10.1200/jco.2007.12.7837 18235113

[77] Madore J Vilain RE Menzies AM Kakavand H Wilmott JS Hyman J. PD-L1 expression in melanoma shows marked heterogeneity within and between patients: implications for anti-PD-1/PD-L1 clinical trials. Pigm Cell Melanoma R. (2014) 28:245–53. doi: 10.1111/pcmr.12340, PMID: 25477049

[78] O'Malley DP Yang Y Boisot S Sudarsanam S Wang JF Chizhevsky V. Immunohistochemical detection of PD-L1 among diverse human neoplasms in a reference laboratory: observations based upon 62,896 cases. Mod Pathol. (2019) 32:929–42. doi: 10.1038/s41379-019-0210-3, PMID: 30760860 PMC6760643

[79] Kaunitz GJ Cottrell TR Lilo M Muthappan V Esandrio J Berry S. Melanoma subtypes demonstrate distinct PD-L1 expression profiles. Lab Investig. (2017) 97:1063–71. doi: 10.1038/labinvest.2017.64, PMID: 28737763 PMC5685163

