# Supplementary material for: Immune checkpoint inhibitors efficacy across solid cancers and the utility of PD-L1 as a biomarker of response: a systematic review and meta-analysis
Source: Front Med (Lausanne). 2023 May 12;10:1192762. doi: 10.3389/fmed.2023.1192762 (PMC10219231; doi:10.3389/fmed.2023.1192762)
Supplement: Supplementary file 1 [file Data_Sheet_1.DOCX]

Supplementary materials:

A systematic review and meta-analysis of checkpoint inhibitor efficacy in solid cancers

TS Fitzsimmons Bsc^1*^, N Singh Bsc^1*^ TDJ Walker PhD^2^, C. Newton MD MRCOG^3^, DGR Evans MD FRCP FRCOG^4^, EJ Crosbie PhD FRCOG^2^, NAJ Ryan PhD MRCS MRCOG^5,6^

1. Clinical Medical School, University of Bristol, Bristol, UK
2. Division of Cancer Sciences, Faculty of Biology, Medicine and Health, University of Manchester, St Mary’s Hospital, Manchester, UK.
3. Department of Obstetrics and Gynaecology, St Michaels Hospital, Bristol, UK
4. Division of Evolution and Genomic Medicine, St Mary’s Hospital, University of Manchester, Manchester, UK
5. The College of Medicine and Veterinary Medicine, University of Edinburgh, Edinburgh, UK
6. Department of Gynaecology Oncology, Royal Infirmary of Edinburgh, Edinburgh, UK

Table of Contents

[1. Search Methods 4](#_Toc125564616)

[1.1.1 MEDLINE search strategy 4](#_Toc125564617)

[1.1.2 PubMed Search Strategy 5](#_Toc125564618)

[1.2 Inclusion criteria 6](#_Toc125564619)

[1.2.1 Types of participants 6](#_Toc125564620)

[1.2.1 Types of intervention 6](#_Toc125564621)

[1.2.1 Types of studies 6](#_Toc125564622)

[1.2.1 Outcomes 6](#_Toc125564623)

[1.3 Extended Statistical methods. 10](#_Toc125564624)

[1.4 Extended results 10](#_Toc125564625)

[2. Subgroup Analysis 19](#_Toc125564626)

[1.5 Subgroup analysis on PD-L1 expression 41](#_Toc125564627)

[Reference: 48](#_Toc125564628)

List of Tables

[Table S1 Inclusion Criteria 7](#_Toc125564694)

[Table S2 Data capture points 8](#_Toc125564695)

[Table S3 Subgroups for analysis 9](#_Toc125564696)

[Table S4 Excluded studies 16](#_Toc125564697)

Table of Figures

[Figure S1 Risk of bias plot for included studies 17](#_Toc125564663)

[Figure S2 Approach to formulating summary assessments of risk of bias for each outcome 18](#_Toc125564664)

[Figure S3 Small Cell vs Non-Small Cell Lung cancer analysis 19](#_Toc125564665)

[Figure S4 Subgroup meta-analysis comparing the efficacy of ICPI use on OS in the primary cancer vs the disease recurrence setting 20](#_Toc125564666)

[Figure S5 Subgroup meta-analysis of ICPIs efficacy on OS in those cancers that responded to routine primary treatment vs those cancers that did not respond to routine primary treatment 21](#_Toc125564667)

[Figure S6 Subgroup meta-analysis of ICPI efficacy on OS taken from studies with in which most cancers expressed PDL1 vs studies in which a minority of cancers expressed PDL1 22](#_Toc125564668)

[Figure S7 Subgroup meta-analysis of ICPI efficacy on OS taken from studies in which a single ICPI vs multiple ICPIs were used. 23](#_Toc125564669)

[Figure S8 Subgroup meta-analysis of ICPI efficacy on OS taken from studies deemed to have low vs high bias. 24](#_Toc125564670)

[Figure S9 Subgroup meta-analysis of ICPI efficacy on OS taken from studies with and without a placebo control arm. 25](#_Toc125564671)

[Figure S10 Meta-analysis of ICPI efficacy on PFS sub-grouped by cancer site 26](#_Toc125564672)

[Figure S11 Meta-analysis of ICPI efficacy on PFS in lung cancer, sub-grouped by small cell and non-small cell pathologies 27](#_Toc125564673)

[Figure S12 Meta-analysis of ICPI efficacy on PFS in studies reporting the primary presentation vs recurrent setting. 28](#_Toc125564674)

[Figure S13 Subgroup meta-analysis of ICPIs efficacy on PFS in those cancers that responded to routine primary treatment vs those cancers that did not respond to routine primary treatment 29](#_Toc125564675)

[Figure S14 Subgroup meta-analysis of ICPI efficacy on PFS taken from studies with in which most cancers expressed PDL1 vs studies in which a minority of cancers expressed PDL1 30](#_Toc125564676)

[Figure S15 : Subgroup meta-analysis comparting the PFS grouped by studies that used different ICPI agents 31](#_Toc125564677)

[Figure S16 Subgroup meta-analysis of ICPI efficacy on PFS taken from studies in which a single ICPI vs multiple ICPIs were used 32](#_Toc125564678)

[Figure S17 Subgroup meta-analysis of ICPI efficacy on PFS taken from studies deemed to have low vs high bias 33](#_Toc125564679)

[Figure S18 Subgroup meta-analysis of ICPI efficacy on PFS taken from studies with and without a placebo control arm. 34](#_Toc125564680)

[Figure S19 Subgroup analysis showing risk of genitourinary side effects ICPI vs standard treatment/placebo 35](#_Toc125564681)

[Figure S20 Subgroup analysis showing risk of gastrointestinal side effects ICPI vs standard treatment/placebo 36](#_Toc125564682)

[Figure S21 Subgroup analysis showing risk of dermatological side effects ICPI vs standard treatment/placebo 37](#_Toc125564683)

[Figure S22 Subgroup analysis showing risk of haematological side effects ICPI vs standard treatment/placebo 38](#_Toc125564684)

[Figure S23 Subgroup analysis showing risk of anaemia with ICPI vs standard treatment/placebo 39](#_Toc125564685)

[Figure S24 Subgroup analysis showing risk of neutropenia and leucopoenia with ICPI vs standard treatment/placebo 39](#_Toc125564686)

[Figure S25 Subgroup analysis showing risk of neurological side effects ICPI vs standard treatment/placebo 40](#_Toc125564687)

[Figure S26 Subgroup analysis showing risk of other side effects ICPI vs standard treatment/placebo 40](#_Toc125564688)

[Figure S27 Subgroup meta-analysis of ICPI efficacy on OS taken from studies in which PD-L1 expression was deemed significant by the authors grouped by study bias 42](#_Toc125564689)

[Figure S28 Subgroup meta-analysis of ICPI efficacy on OS taken from studies in which PD-L1 expression was deemed significant by the authors 43](#_Toc125564690)

[Figure S29 Subgroup meta-analysis of ICPI efficacy on PFS taken from studies in which PD-L1 expression was deemed significant by the authors 43](#_Toc125564691)

[Figure S30 Subgroup meta-analysis of ICPI efficacy on OS displayed by cancer site taken from studies in which PD-L1 expression was deemed significant by the authors 44](#_Toc125564692)

[Figure S31 Subgroup meta-analysis of ICPI efficacy on OS displayed by primary cancer and disease recurrence from studies in which PD-L1 expression was deemed significant by the authors 45](#_Toc125564693)

# Search Methods

## 1.1.1 MEDLINE search strategy

1. randomizedcontrolledtrial.pt.
2. controlledclinicaltrial.pt.
3. randomized.ab.
4. placebo.ab.
5. drugtherapy.fs.
6. randomly.ab.
7. trial.ab.
8. groups.ab
9. 1 or 2 or 3 or 4 or 5 or 6 or 7 or 8
10. (animals not (humans and animals)).sh.
11. 9not10
12. (cancer* or carcinoma*or neoplasm* or tumor*or tumour*or malignan*).mp.
13. exp DNA Mismatch repair
14. exp DNA Mismatch
15. DNA repair.mp.
16. exp "Check point inhibition"
17. (PD*).mp.
18. (PD-*).mp.
19. (pembrolizumab or keytruda).mp.
20. (Nivolumab or opdivo).mp.
21. (cemiplimab or libtayo).mp.
22. (Atezolizumab or tecentriq or MPDL3280A).mp.
23. (Durvalumab or Imfinzi).mp
24. 13 or 14 or 15 or 16 or 17 or 18 or 19 or 20 or 21 or 22 or 23
25. 11 and 12 and 24

Key: ab=abstract fs=floating subheading sh=subject heading mp=title, abstract, subject headings, heading word, drug trade name, original title, device manufacturer, drug manufacturer name

## 1.1.2 PubMed Search Strategy

(((((((((((((((((((((((((Programmed Cell Death 1 Receptor[MeSH Terms]) OR PD-1 Receptor[Title/Abstract]) OR PD1 Receptor[Title/Abstract]) OR Receptor, PD- 1[Title/Abstract]) OR Programmed Cell Death 1 Protein[Title/Abstract]) OR Programmed death receptor 1[Title/Abstract]) OR PD-1[Title/Abstract]) OR Programmed death ligand1[Title/Abstract]) OR PD-L1[Title/Abstract]) OR Programmed Cell Death 1 Receptor[Title/Abstract]) OR nivolumab[Title/Abstract]) OR pembrolizumab[Title/Abstract]) OR atezolizumab[Title/Abstract]) OR durvalumab[Title/Abstract]) OR cemiplimab[Title/Abstract]) OR toripalimab[Title/Abstract]) OR sintilimab[Title/Abstract]) OR avelumab[Title/Abstract]) OR Keytruda[Title/Abstract]) OR Opdivo[Title/Abstract]) OR Libtayo[Title/Abstract]) OR Tecentriq[Title/Abstract]) OR Bavencio[Title/Abstract]) OR Imfinzi[Title/Abstract]) OR Camrelizumab[Title/Abstract]) AND ((((((((((((((((((((((((((Neoplasms[MeSH Terms]) OR Neoplasm*[Title/Abstract]) OR tumor*[Title/Abstract]) OR Cancer*[Title/Abstract]) OR malignan*[Title/Abstract]) OR carcinoma*[Title/Abstract]) OR tumour*[Title/Abstract]) OR adenocarcin*[Title/Abstract]) OR Hematologic Neoplasms[MeSH Terms]) OR hematolog* malignan*[Title/Abstract]) OR hematolog* neoplas*[Title/Abstract]) OR lymphom*[Title/Abstract]) OR leukem*[Title/Abstract]) OR hodgkin*[Title/Abstract]) OR lymphogranulomato*[Title/Abstract]) OR histiocy*[Title/Abstract]) OR granulom*[Title/Abstract]) OR non- hodgkin*[Title/Abstract]) OR nonhodgkin*[Title/Abstract]) OR lymphosarcom*[Title/Abstract]) OR reticulosarcom*[Title/Abstract]) OR brill- symmer*[Title/Abstract]) OR plasmacytom*[Title/Abstract]) OR myelom*[Title/Abstract]) OR sezary[Title/Abstract]) OR myelodysplas*[Title/Abstract]) OR aplastic anem*[Title/Abstract]) AND (((((((((((randomized controlled trial[Publication Type]) OR controlled clinical trial[Publication Type]) OR randomized[Title/Abstract]) OR placebo[Title/Abstract]) OR randomly[Title/Abstract]) OR trial[Title/Abstract]) OR groups[Title/Abstract])) NOT ((animals[MeSH Terms]) NOT ((humans[MeSH Terms]) AND animals[MeSH Terms]))))))

## 1.2 Inclusion criteria

### 1.2.1 Types of participants

Women and men ≥ 18 years old with solid cancers in whom ICPIs have been used. Individuals with other concurrent malignancies have been excluded. We excluded melanomas and haematological cancers.

### 1.2.1 Types of intervention

- Immune checkpoint inhibitors versus no treatment
- Immune checkpoint inhibitors + conventional chemotherapy versus conventional chemotherapy
- Immune checkpoint inhibitors versus conventional chemotherapy

### 1.2.1 Types of studies

Only phase III, randomised control trials (RCTs) were selected.

### 1.2.1 Outcomes

The primary outcome of this review was Overall Survival (OS). The secondary outcomes were Progression Free Survival (PFS), Objective Response Rate (ORR), Quality of Life (QoL) and side effects.

| **Inclusion Criteria** | **Exclusion Criteria** |
| --- | --- |
| - Phase III RCT with > 50 participants - Use a population of women and men aged ≥ 18 years old with solid cancers - Intervention of ICPIs (either anti-PD-1 therapy or anti-PD-L1 therapy) - Use of the following comparators:   - No treatment or placebo   - Conventional chemotherapy/ SOC chemotherapy - Report the following as outcomes:   - Overall survival (OS)   - Progression free survival (PFS)   - Serious adverse events | - Phase I or II RCTs - Case reports, review articles, animal or in vitro studies - Participants with concurrent malignancies - Intervention group receiving other biological agents which are not ICPIs - Melanomas - Full text not available |

Table S1 Inclusion Criteria

|  | **Outcomes or data extracted** |
| --- | --- |
| **Study characteristics** | - First author - Publication date - Study start date - Setting/ country - Mean follow up period - Treatment characteristics for both intervention and control (drug, dosage, frequency) - Number of participants - Mean age of participants - Number of PD-1/PD-L1 participants |
| **Time to event data** | OS and PFS   - Hazard ratios (HR), 95% confidence intervals (CI) and p-values were collected on an intention to treat basis.   Where studies reported overall OS/ PFS in all participants **AND** OS/PFS for PD-1/PD-L1 positive participants, this data was also extracted for further broad group analysis of OS and PFS. This data was referred to as PD-L1 studies. |
| **Categorical data** | Serious adverse events (G>3) sorted by category:   - Haematological - Neurological - Genitourinary - Gastrointestinal - Dermatological - Other   The number of each event occurring within these categories was recorded for the control and intervention group, alongside the number of patients within the two treatment groups. From this risk ratios (RR) and 95% CIs were calculated for each category of serious adverse events within each category. |

Table S2 Data capture points

| **Subgroup** | **Definition** |
| --- | --- |
| Advanced disease | High stage disease (>Stage II) |
| Recurrent Disease | Disease which recurred following treatment |
| Majority PD-L1 positivity | >50% of the participants having PD-L1 positive tumours |
| Minority PD-L1 positivity | <50% of the participants having PD-L1 positive tumours |
| PD-L1 positive | Studies where outcomes were reported for participants with TPS >1% |
| Primary treatment failure | Disease progression despite having received the primary treatment for cancer |
| No primary treatment failure | No primary treatment given to participants |
| Placebo use | Studies where control group received a placebo |
| No placebo use | Studies where the control group received treatments which were not a placebo (e.g. SOC chemotherapy) |
| Low bias rating | Studies with 4 or more domains assessed as low bias |
| High bias rating | Studies not assessed as low bias |

Table S3 Subgroups for analysis

## 1.3 Extended Statistical methods.

Random-effects models between-study heterogeneity was described by both tau-squared τ^2^ with Sidik-Jonkman estimator and by Higgins & Thompson’s Heterogeneity *I^2^* score (Low: 25%, 50%: moderate, 75% low heterogeneity).^1^ HR pools inverse variance methods were adopted by Random Effects (RE).

Small study effects and inferred risk of publication bias due to effects sizes were statistically inspected by contour funnel plot asymmetry and Egger’s tests while inferred risk of publication bias due to p value bias were inspected by p-curves.^2^ Influence analysis using leave-one-out methods determined influential studies as inspected by Baujat plots and forest plots with studies ordered by overall effect size or by *I^2^*. ^3^

HR and RRs for individual and pooled studies were displayed in forest plots, with reference to their 95% CIs, and relative weighting within the meta-analysis. Sensitivity analysis was conducted to exclude studies at high risk of bias and unadjusted results. Subgroup analysis was carried out to explore heterogeneity and was themed around clinically relevant scenarios. In addition, these subgroups enabled specific clinical questions to be examined. These subgroups are descried in detail in Table S3.

## 1.4 Extended results

*Analysis power, small study effects, and influential studies*

Every heterogeneity-graded power analysis predicted random effects meta-analyses would be sufficiently powered above 85% for our study size. Time-to-event overall survival values were 97% (low heterogeneity); 95% (medium heterogeneity); and 91% (high heterogeneity). Time-to-event progression free survival values were 97% (low heterogeneity); 94% (medium heterogeneity); and 90% (high heterogeneity).

Small study effects moderate biases were evident on funnel plots as anticipated. No studies presented outliers with respect to large Hedge’s g value with low standard error. P-curve assessment indicated that both OS and PFS analyses contain evidential data, and that evidential data was neither absent nor inadequate. Binomial, full curve, and half curve p values were p>0.99 (Right-Skewness tests) and p<0.001 (Flatness Tests) for OS and PFS analyses.

OS random effects influence analysis Baujat plots highlighted a single study (Vokes *et al.,* 2018)^4^ that contributed both large heterogeneity and influence on the pooled result, while Burtness *et al.,* 2019^5^ contributed large heterogeneity with minimal influence. PFS random-effects influence analysis Baujat plots depicted a single study (Fennell *et al*., 2021)^6^ that contributed small heterogeneity with large influence on the pooled result. Rudin et al., 2020^7^ contributed large heterogeneity with minimal influence. The funnel plots, hedges values and Bauiat plots are available on request.

*Overall Survival Disease specific characteristics*

Further subgroup analysis was performed comparing ICPIs efficacy in cancers that had a meaningful clinical response (as defined by the study authors) to routine primary treatment vs those cancer that had failed to respond to routine primary treatment. Here again, ICPIs were efficacious in both settings: primary treatment response OS HR 0.76 (95% CI 0.70 to 0.83), no primary treatment response OS HR 0.73 (95% CI 0.68 to 0.79). These data are shown in Figure S4.

Interestingly, subgroup analysis comparing studies in which most cancers demonstrated significant PD-L1 expression vs those studies in which a minority of cancer demonstrated significant PD-L1 expression reported similar effect of ICIP use on OS. In the minority PD-L1 cohort HR 0.73 (95% CI 0.68 to 0.78) vs majority PD-L1 cohort HR 0.76 (95% CI 0.70 to 0.84). This difference did not reach significance (p=0.42). This was maintained even when studies exploring the same cancer site were directly compared. These data are shown in Figure S5.

*Side effect Profile*

The use of ICPIs did not lead to a significant difference in any genitourinary, gastrointestinal, dermatological, neurological side effects when compared with standard treatment. Dermatological subgroup analysis is of limited utility as only a few studies reported these symptoms. Of note, there was a significant reduction in haematological side effects with the use of ICPIs (RR 0.36 (95% CI 0.25 to 0.53)) with this being driven by lower rates of neutropoenia and anaemia (See Figures S18-24). Furthermore, there was no significant difference in the risk of patients reporting dyspnoea, fatigue, haemoptysis, pulmonary embolus, pyrexia, or respiratory failure (Figure S25).

| Title | **Author or NCT number** | **Year** | **Reason for exclusion** |
| --- | --- | --- | --- |
| Predictive correlates of response to the anti-PD-L1 antibody MPDL3280A in cancer patients | Roy S Herbst et al | 2014 | No data |
| Camrelizumab in advanced or metastatic solid tumour patients with DNA mismatch repair deficient or microsatellite instability high: an open-label prospective pivotal trial | [Jingde Chen et al](javascript:;) | 2020 | Wrong study design |
| Nivolumab plus Ipilimumab in Lung Cancer with a High Tumor Mutational Burden | Hellman et al | 2018 | Wrong study design |
| Exposure to anti-PD-1 causes functional differences in tumor-infiltrating lymphocytes in rare solid tumors | [Caitlin A. Creasy et al](https://onlinelibrary.wiley.com/action/doSearch?ContribAuthorStored=Creasy%2C+Caitlin+A) | 2019 | Background article |
| Efficacy of Pembrolizumab in Patients with Noncolorectal High Microsatellite Instability/Mismatch Repair-Deficient Cancer: Results from the Phase II KEYNOTE-158 Study | [Aurelien Marabelle et al](https://pubmed.ncbi.nlm.nih.gov/?term=Marabelle+A&cauthor_id=31682550) | 2020 | Wrong study design |
| Mismatch repair deficiency predicts response of solid tumors to PD-1 blockade | Dung T. Le et al | 2017 | Wrong study design |
| PD-1 Blockade in Tumors with Mismatch-Repair Deficiency | [Yoshinari Asaoka et al](https://pubmed.ncbi.nlm.nih.gov/?term=Asaoka+Y&cauthor_id=26559583) | 2015 | Wrong study design |
| PD-1 Blockade in Tumors with Mismatch-Repair Deficiency | Dung T. Le et al | 2015 | Wrong study design |
| ASCO Reveals Additional Promising Results with Immunotherapies | [Vicki Brower](javascript:;) | 2015 | Background article |
| Mismatch repair deficiency predicts benefit of anti-PD-1 therapy | [Esther Lau](https://www.thelancet.com/journals/lanonc/article/PIIS1470-2045(15)00031-5/fulltext) | 2015 | Review article |
| A phase 3, randomized, open-label study of epacadostat plus pembrolizumab, pembrolizumab monotherapy, and the EXTREME regimen as first-line treatment for recurrent/metastatic head and neck squamous cell carcinoma (R/M SCCHN): ECHO-304/KEYNOTE-669 | Cohen et al | 2018 | Wrong study design |
| Pembrolizumab Plus Epacadostat vs Pembrolizumab Plus Placebo in Metastatic Non-Small Cell Lung Cancer (KEYNOTE-654-05/ECHO-305-05 | Awad et al | 2018 | Wrong study design |
| Safety and Efficacy of Bevacizumab Plus Standard-of-Care Treatment Beyond Disease Progression in Patients with Advanced Non-Small Cell Lung Cancer: The AvaALL Randomized Clinical Trial | Gridelli et al | 2018 | Wrong study design |
| Safety and Efficacy Study of CC-486 With MK-3475 to Treat Lsafety and efficacy  cally Advanced or Metastatic Non-small Cell Lung Cancer | NCT02546986 | 2015 | Wrong study design |
| Cost-effectiveness of immune checkpoint inhibitors for microsatellite instability-high/mismatch repair-deficient metastatic colorectal cancer | Chu et al | 2018 | Review article |
| FDA Approval Summary: Pembrolizumab for the Treatment of Microsatellite Instability-High Solid Tumors. | Marcus et al | 2019 | Background article |
| Neoadjuvant immunotherapy leads to pathological responses in MMR-proficient and MMR-deficient early-stage colon cancers | Chalabi et al | 2020 | No data |
| Safety, activity, and immune correlates of anti-PD-1 antibody in cancer | Topalian et al | 2012 | Wrong study design |
| Safety of Nivolumab plus Low-Dose Ipilimumab in Previously Treated Microsatellite Instability-High/Mismatch Repair-Deficient Metastatic Colorectal Cancer | Morse et al | 2019 | Wrong study design |
| Pembrolizumab in Combination with Epacadostat or Placebo in Cisplatin-ineligible Urothelial Carcinoma (KEYNOTE-672/ECHO-307) | Balar et al | 2020 | Wrong study design |
| Pembrolizumab + Epacadostat vs Pembrolizumab + Placebo in Recurrent or Progressive Metastatic Urothelial Carcinoma | NCT03374488 | 2020 | Wrong study design |
| PD-1 inhibition in metastatic dMMR/MSI-H colorectal cancer | Sclafani et al | 2017 | Background article |
| Durable Clinical Benefit with Nivolumab Plus Ipilimumab in DNA Mismatch Repair-Deficient/Microsatellite Instability-High Metastatic Colorectal Cancer | Overman et al | 2018 | No data |
| Safety and Antitumor Activity of Anti-PD-1 Antibody, Nivolumab, in Patients with Platinum-Resistant Ovarian Cancer | Hamanishi et al | 2015 | Small sample size |
| Pembrolizumab for Treatment-Refractory Metastatic Castration-Resistant Prostate Cancer: Multicohort, Open-Label Phase II KEYNOTE-199 Study | Antonarikis et al | 2019 | Wrong study design |
| Pembrolizumab in patients with programmed death ligand 1-positive advanced ovarian cancer: Analysis of KEYNOTE-028 | Varga et al | 2019 | Small sample size |
| Analysis of the Prevalence of Microsatellite Instability in Prostate Cancer and Response to Immune Checkpoint Blockade | Abida et al | 2019 | Wrong study design |
| Nivolumab Is Effective in Mismatch Repair–Deficient Noncolorectal Cancers: Results From Arm Z1D—A Subprotocol of the NCI-MATCH (EAY131) Study | Azad et al | 2019 | Wrong study design |
| Study of Pembrolizumab (MK-3475) in Participants with Advanced Solid Tumors (MK-3475-012/KEYNOTE-012) | NCT01848834 | 2020 | Wrong study design |
| A Study of Atezolizumab in Participants with Locally Advanced or Metastatic Urothelial Bladder Cancer (Cohort 2) | NCT02108652 | 2021 | Wrong study design |
| Study of Pembrolizumab (MK-3475) in Participants with Metastatic Castration-Resistant Prostate Cancer (mCRPC)(MK-3475-199/KEYNOTE-199) | NCT01905657 | 2019 | No data |
| Long-Term Outcomes and Retreatment Among Patients with Previously Treated, Programmed Death-Ligand 1‒Positive, Advanced Non‒Small-Cell Lung Cancer in the KEYNOTE-010 Study | Herbst et al | 2020 | Wrong study design |
| A Study Comparing the Efficacy and Safety Between IBI308 and Docetaxel in Patients with Advanced or Metastatic NSCLC | NCT03150875 | 2021 | Wrong study design |
| CheckMate 025 Randomized Phase 3 Study: Outcomes by Key Baseline Factors and Prior Therapy for Nivolumab Versus Everolimus in Advanced Renal Cell Carcinoma | Escudier et al | 2017 | Wrong study design |
| Adjuvant Nivolumab in Resected Esophageal or Gastroesophageal Junction Cancer | Kelly et al | 2021 | Wrong study design |
| Chemotherapy with or without avelumab followed by avelumab maintenance versus chemotherapy alone in patients with previously untreated epithelial ovarian cancer (JAVELIN Ovarian 100): an open-label, randomised, phase 3 trial | Monk et al | 2021 | Wrong study design |
| Adjuvant atezolizumab after adjuvant chemotherapy in resected stage IB–IIIA non-small-cell lung cancer (IMpower010): a randomised, multicentre, open-label, phase 3 trial | Felip et al | 2021 | Wrong study design |
| Pembrolizumab for Persistent, Recurrent, or Metastatic Cervical Cancer | Colombo et al | 2021 | Wrong study design |
| Survival Outcomes in Patients with Previously Untreated BRAF Wild-Type Advanced Melanoma Treated With Nivolumab Therapy: Three-Year Follow-up of a Randomized Phase 3 Trial | Ascierto et al | 2019 | Wrong study design |
| Atezolizumab with or without cobimetinib versus regorafenib in previously treated metastatic colorectal cancer (IMblaze370): a multicentre, open-label, phase 3, randomised, controlled trial | Eng et al | 2019 | Wrong study design |
| Final analysis of a randomised trial comparing pembrolizumab versus investigator-choice chemotherapy for ipilimumab-refractory advanced melanoma | Hamid et al | 2017 | Wrong study design |
| Long-term outcomes in patients with advanced melanoma who had initial stable disease with pembrolizumab in KEYNOTE-001 and KEYNOTE-006 | Hamid et al | 2021 | Wrong study design |
| Atezolizumab plus bevacizumab versus sunitinib in patients with previously untreated metastatic renal cell carcinoma (IMmotion151): a multicentre, open-label, phase 3, randomised controlled trial | Rini et al | 2019 | Wrong study design |
| Pembrolizumab plus Axitinib versus Sunitinib for Advanced Renal-Cell Carcinoma | Rini et al | 2019 | Wrong study design |

Table S4 Excluded studies

Figure S1 Risk of bias plot for included studies


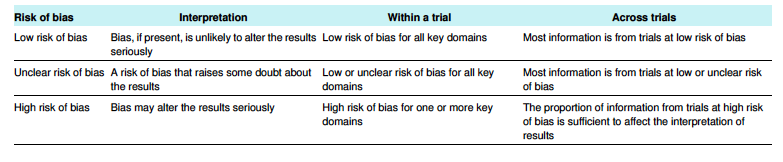


Figure S2 Approach to formulating summary assessments of risk of bias for each outcome

Taken from: Higgins J P T, Altman D G, GÃ¸tzsche P C, JÃ¼ni P, Moher D, Oxman A D et al. The Cochrane Collaboration’s tool for assessing risk of bias in randomised trials BMJ 2011; 343 :d5928 doi:10.1136/bmj.d5928

# Subgroup Analysis


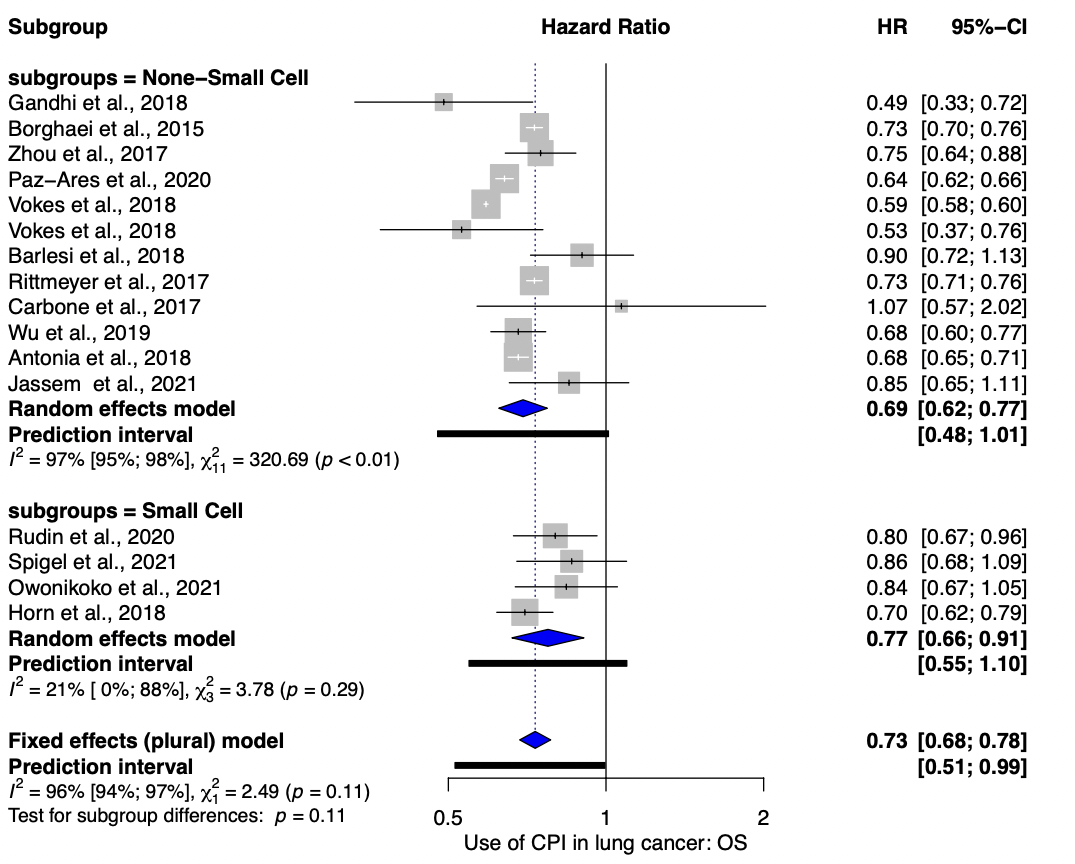


Figure S3 Small Cell vs Non-Small Cell Lung cancer analysis


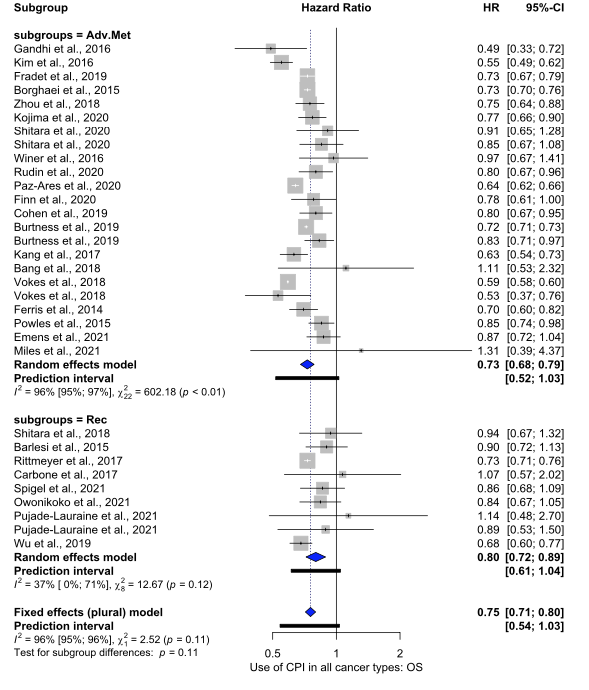


Figure S4 Subgroup meta-analysis comparing the efficacy of ICPI use on OS in the primary cancer vs the disease recurrence setting

Figure S5 Subgroup meta-analysis of ICPIs efficacy on OS in those cancers that responded to routine primary treatment vs those cancers that did not respond to routine primary treatment

Figure S6 Subgroup meta-analysis of ICPI efficacy on OS taken from studies with in which most cancers expressed PDL1 vs studies in which a minority of cancers expressed PDL1


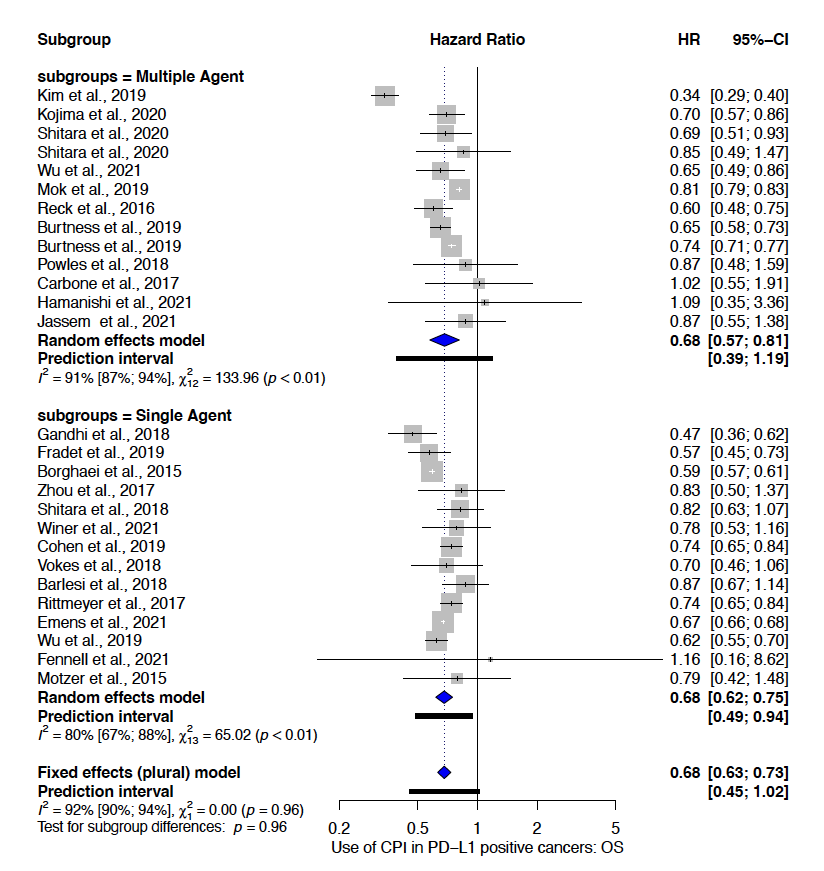


Figure S7 Subgroup meta-analysis of ICPI efficacy on OS taken from studies in which a single ICPI vs multiple ICPIs were used.


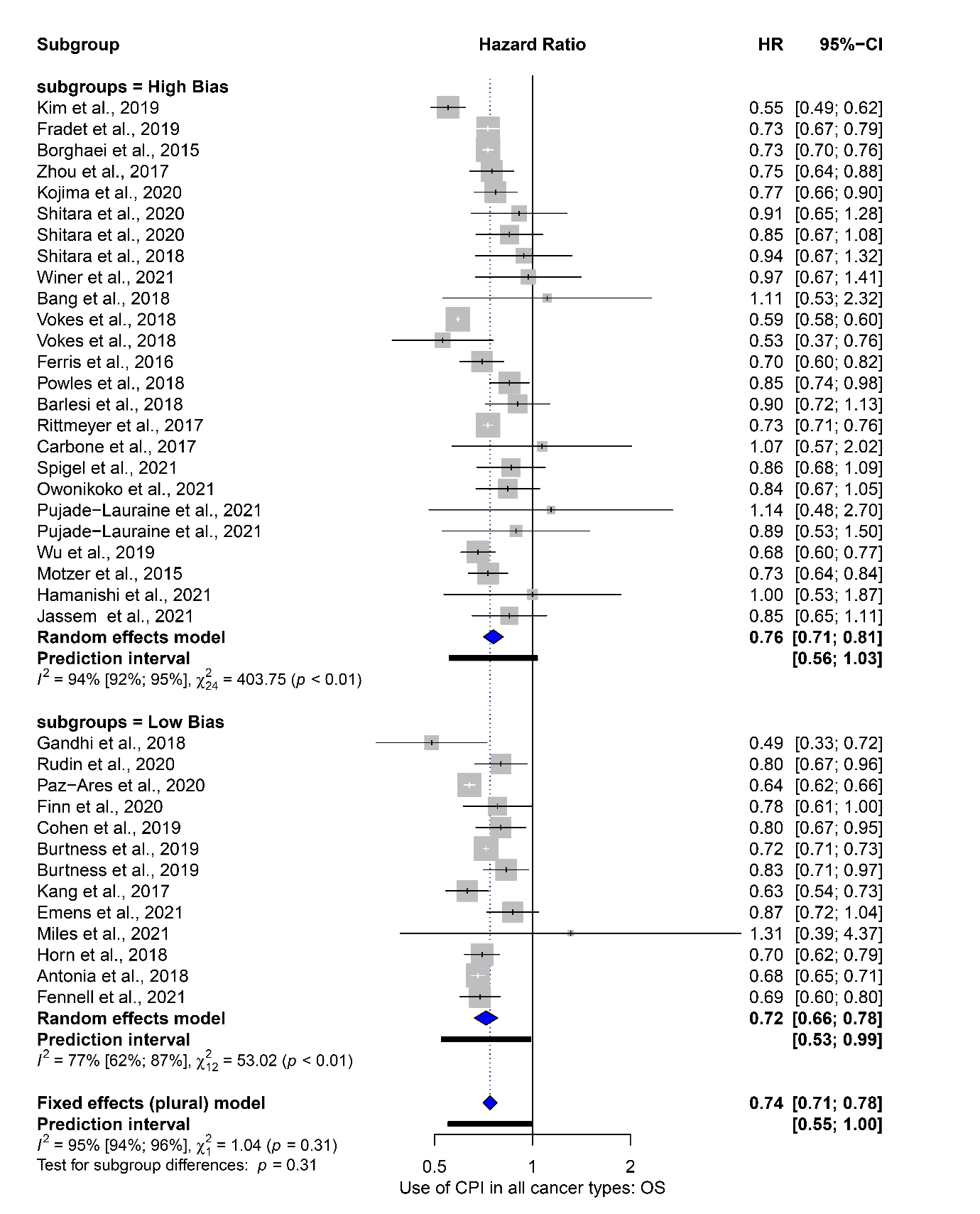
Figure S8 Subgroup meta-analysis of ICPI efficacy on OS taken from studies deemed to have low vs high bias.


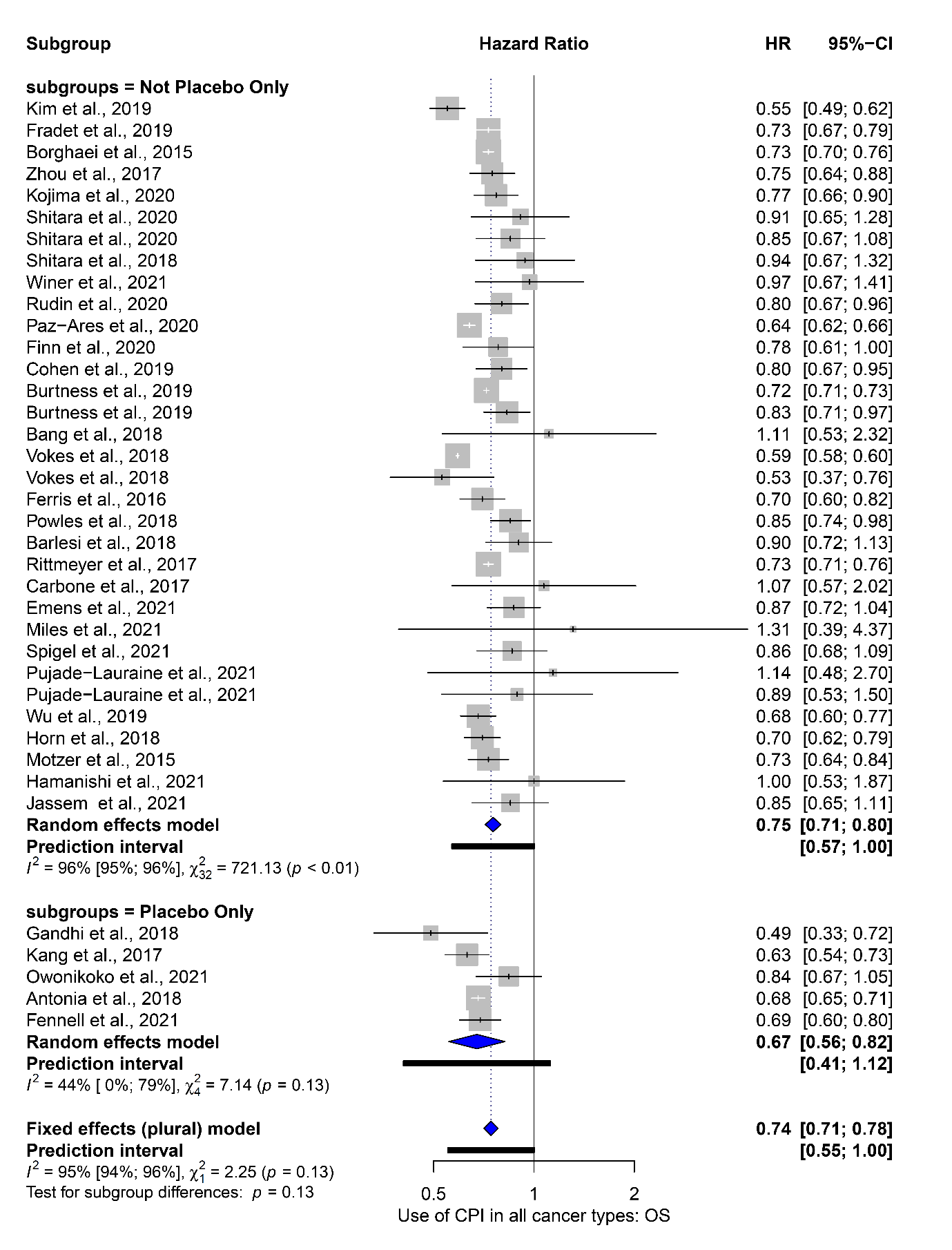


Figure S9 Subgroup meta-analysis of ICPI efficacy on OS taken from studies with and without a placebo control arm.


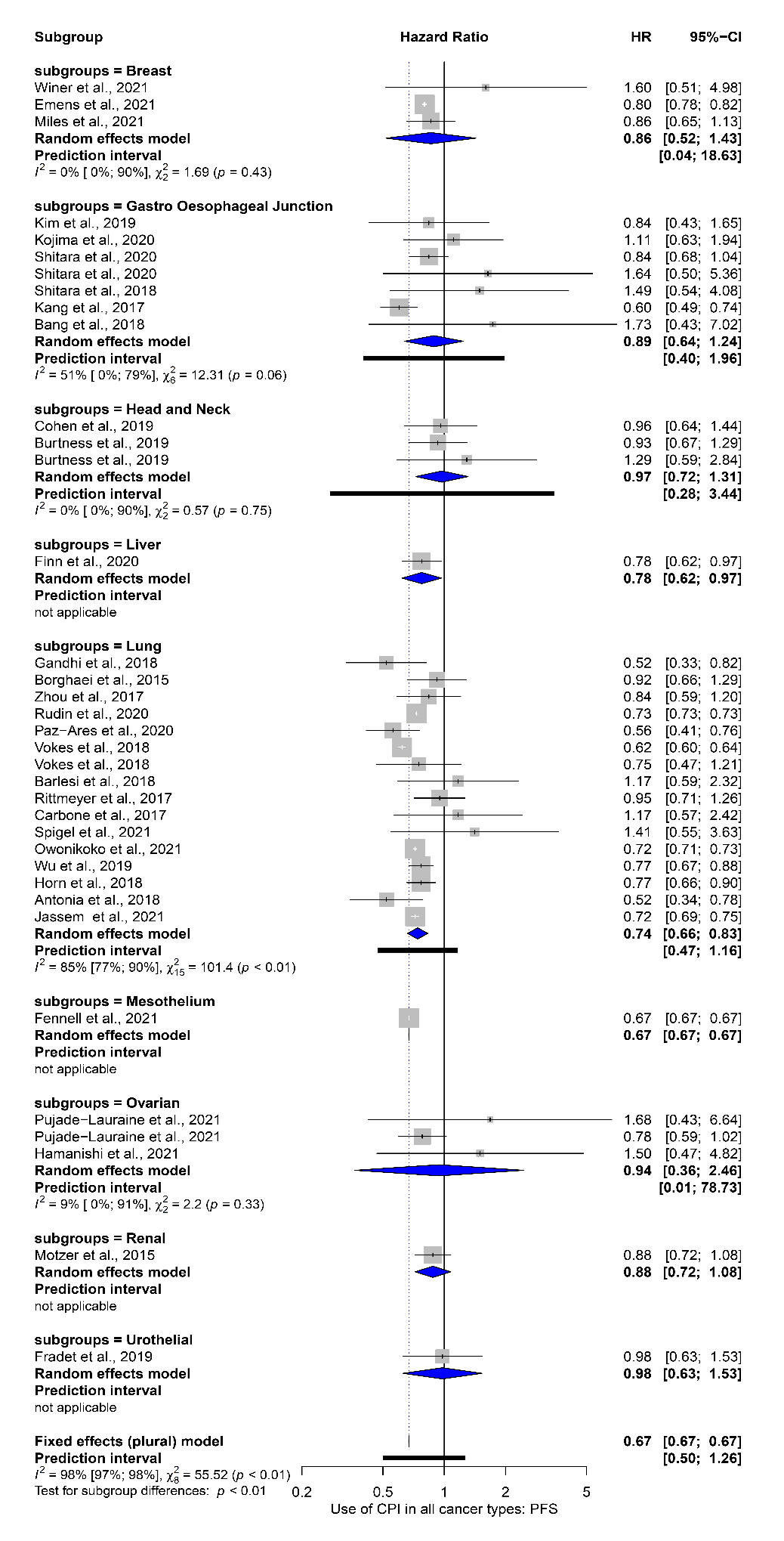
Figure S10 Meta-analysis of ICPI efficacy on PFS sub-grouped by cancer site


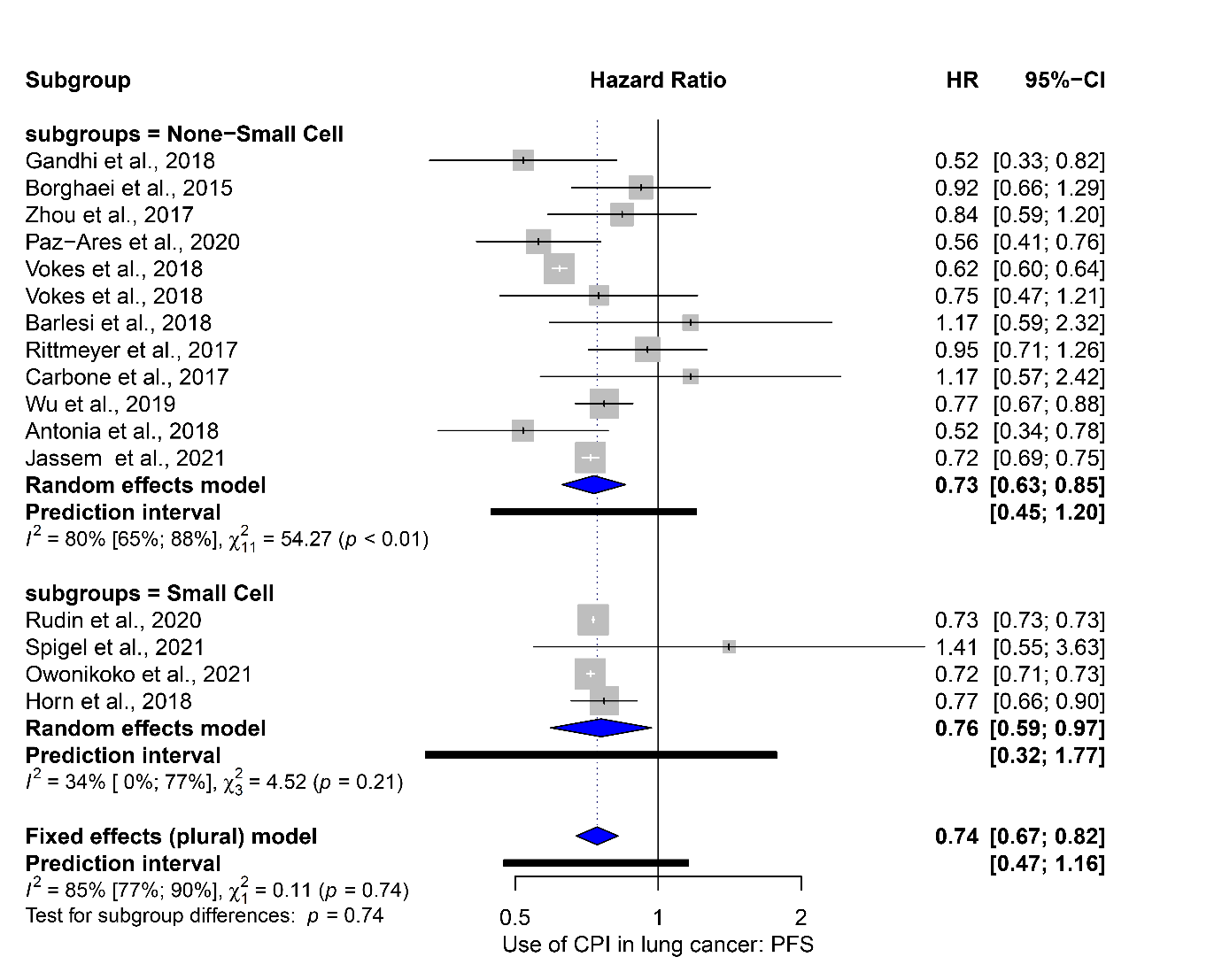


Figure S11 Meta-analysis of ICPI efficacy on PFS in lung cancer, sub-grouped by small cell and non-small cell pathologies


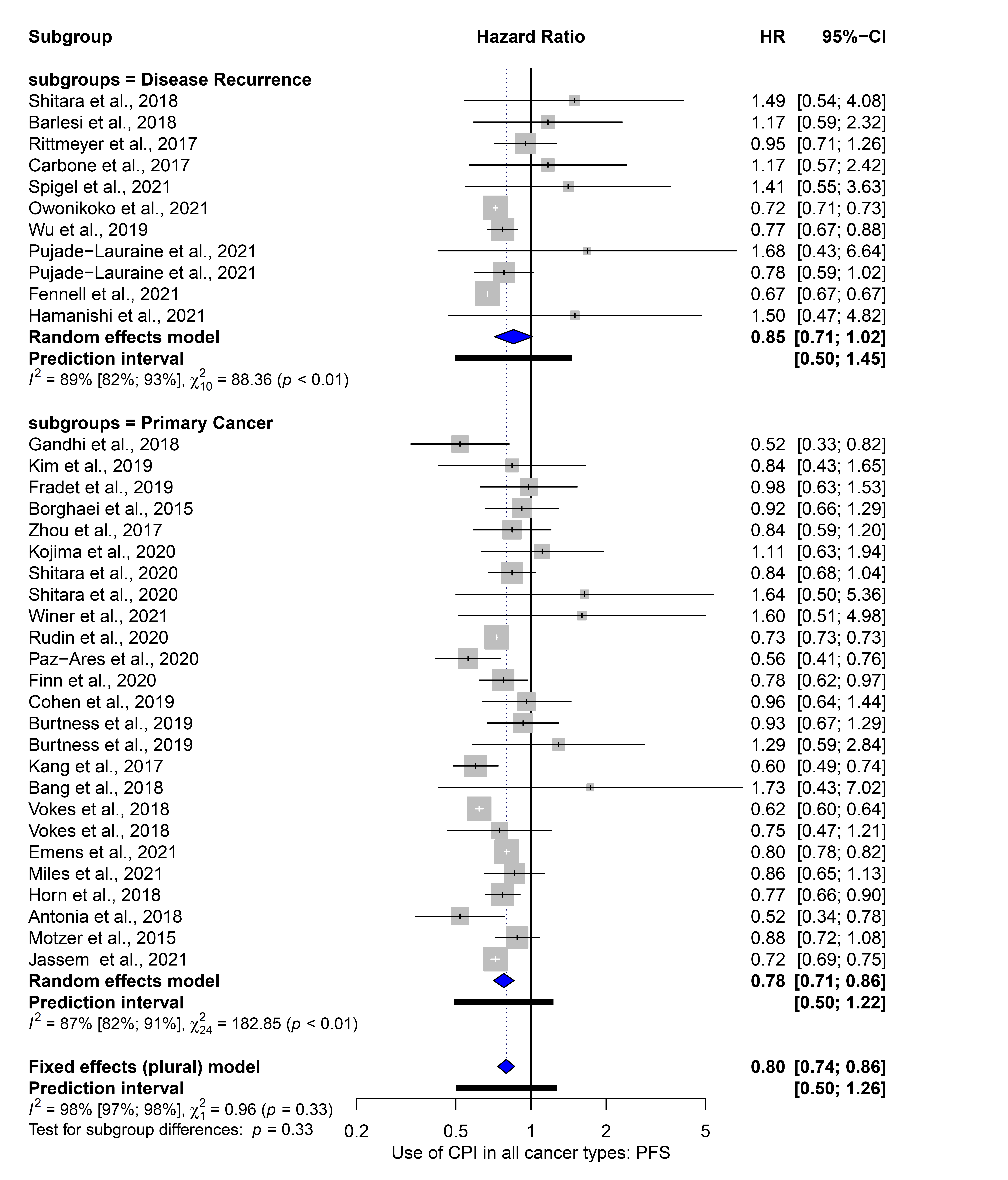


Figure S12 Meta-analysis of ICPI efficacy on PFS in studies reporting the primary presentation vs recurrent setting.


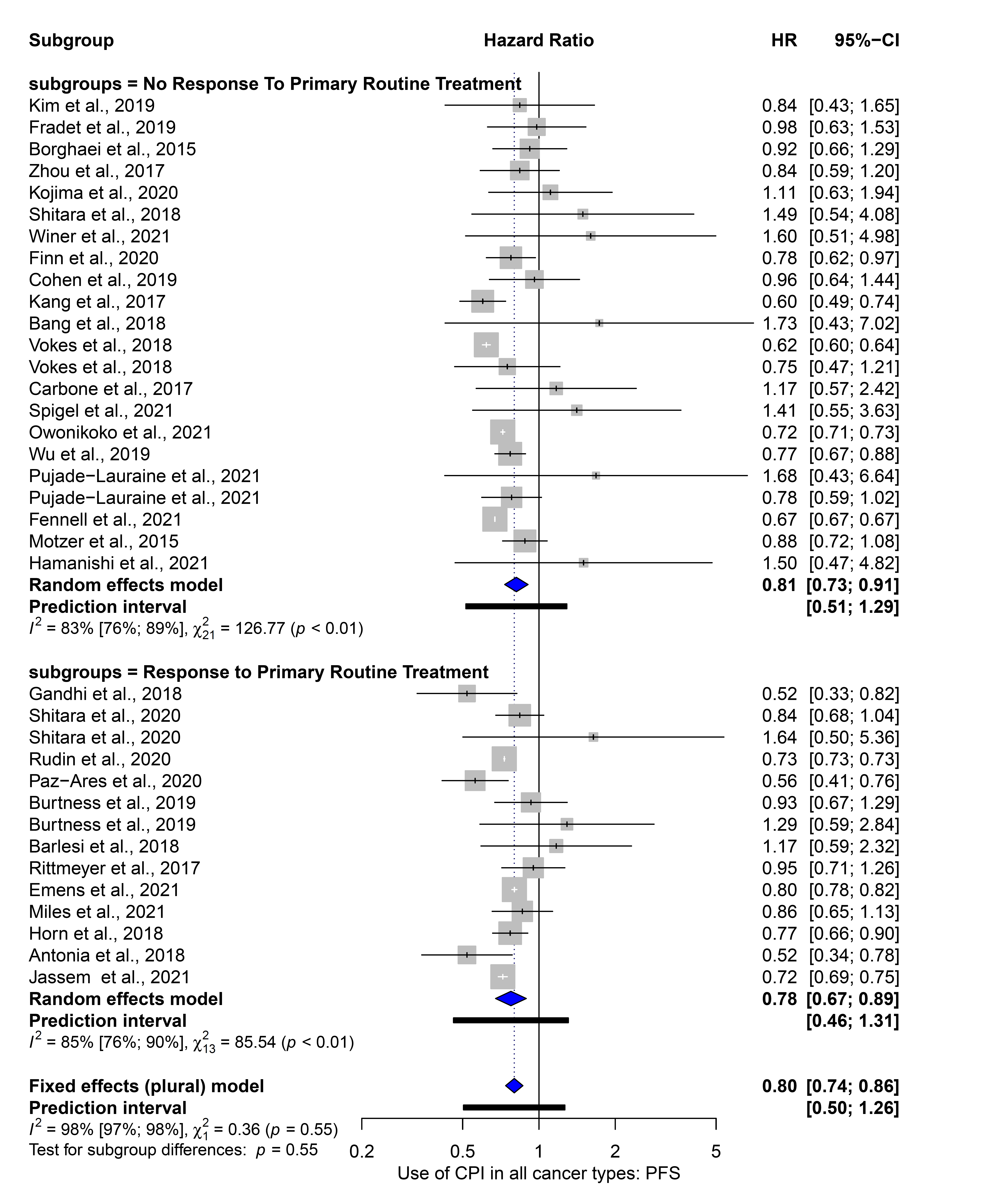


Figure S13 Subgroup meta-analysis of ICPIs efficacy on PFS in those cancers that responded to routine primary treatment vs those cancers that did not respond to routine primary treatment


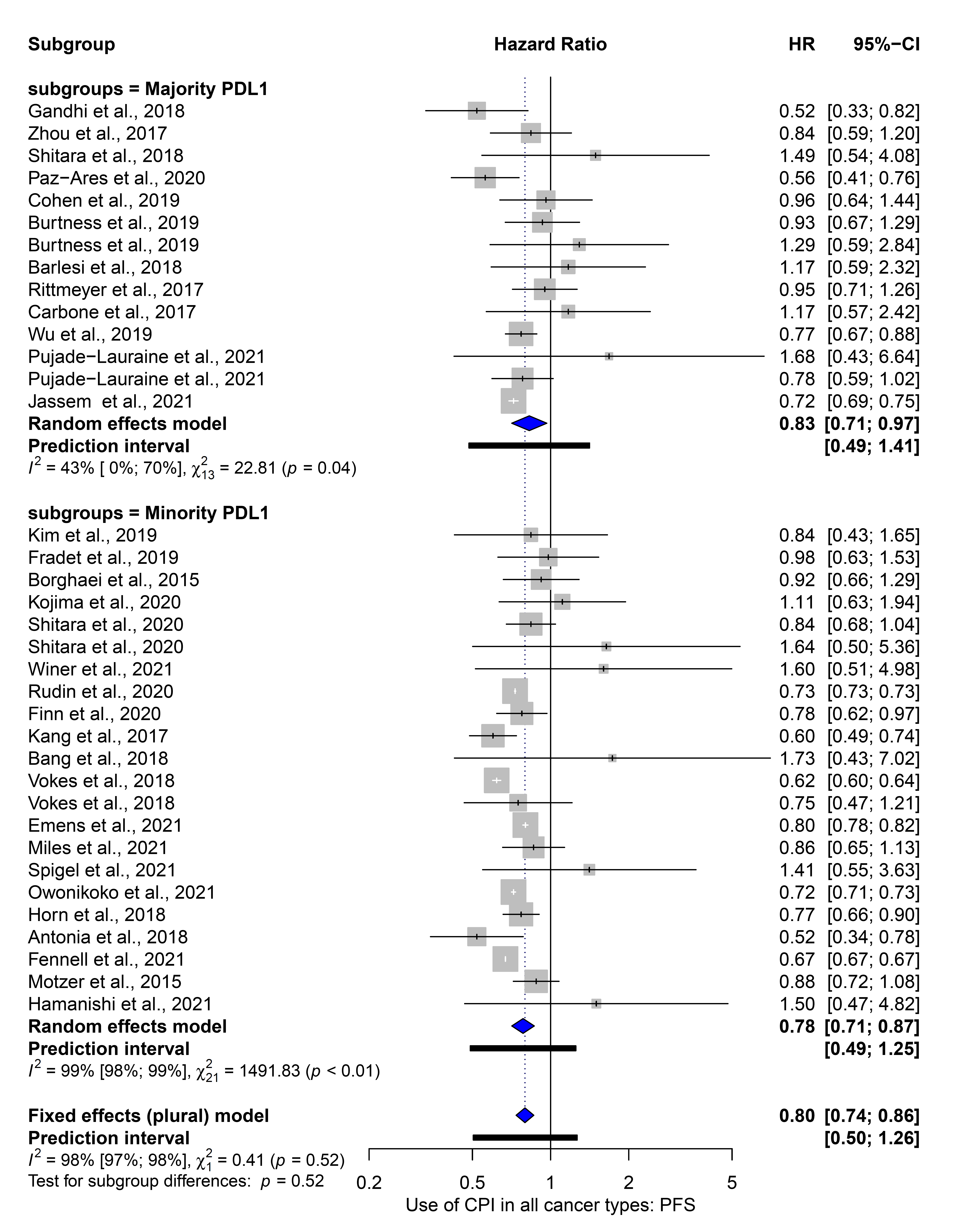


Figure S14 Subgroup meta-analysis of ICPI efficacy on PFS taken from studies with in which most cancers expressed PDL1 vs studies in which a minority of cancers expressed PDL1


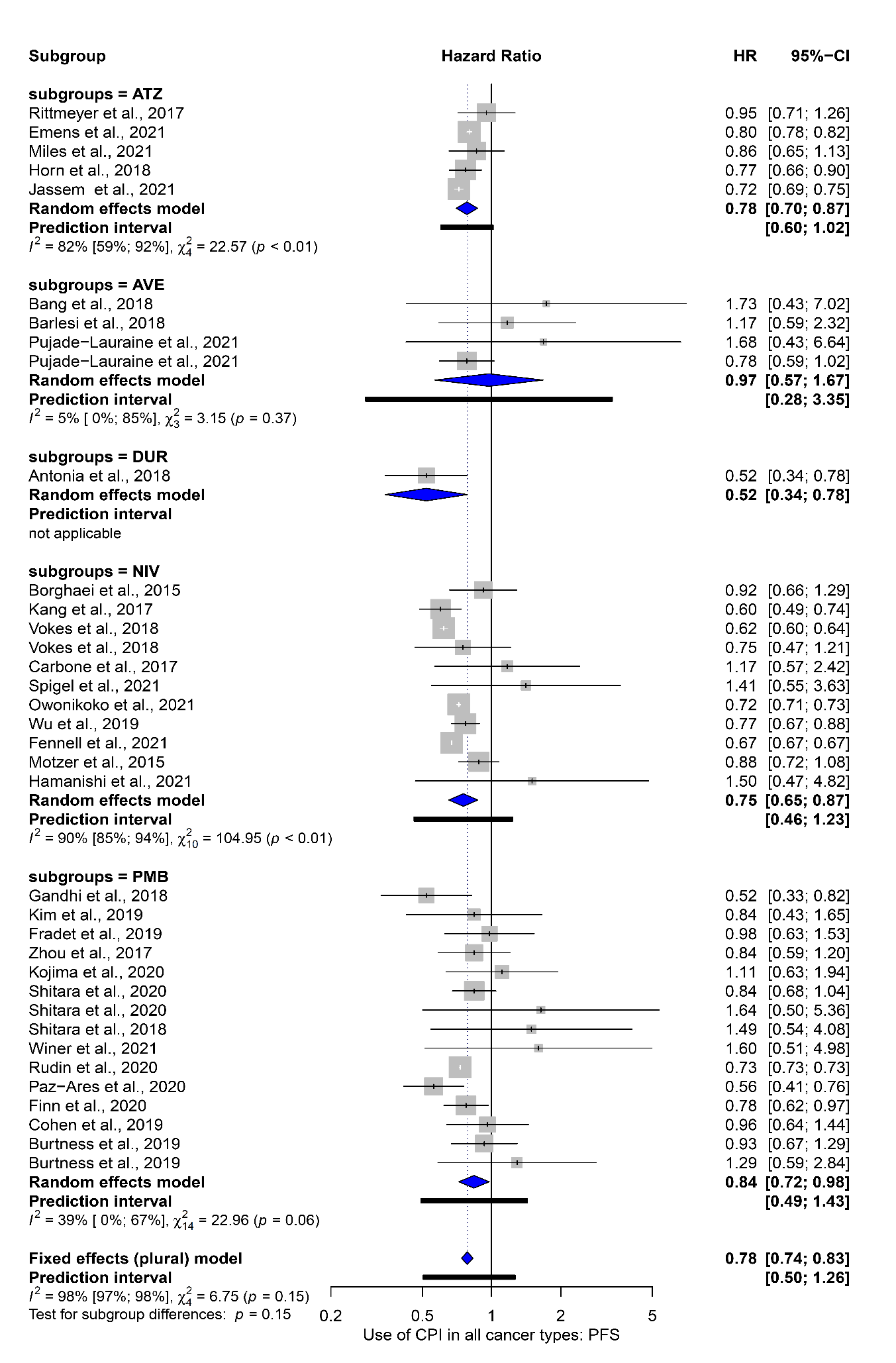
Figure S15 : Subgroup meta-analysis comparting the PFS grouped by studies that used different ICPI agents


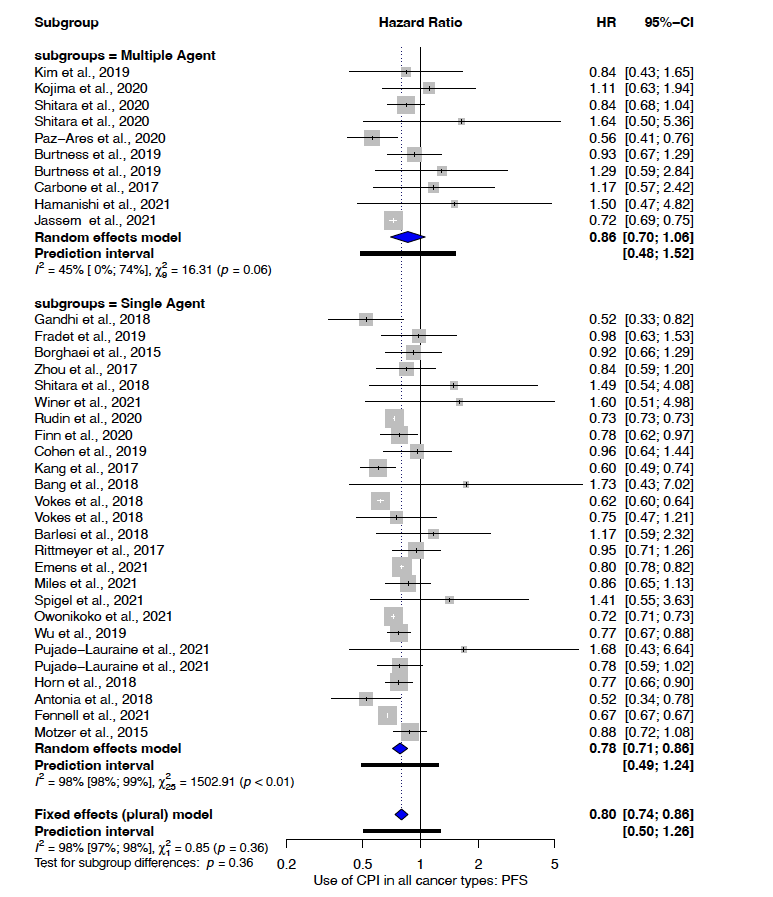


Figure S16 Subgroup meta-analysis of ICPI efficacy on PFS taken from studies in which a single ICPI vs multiple ICPIs were used


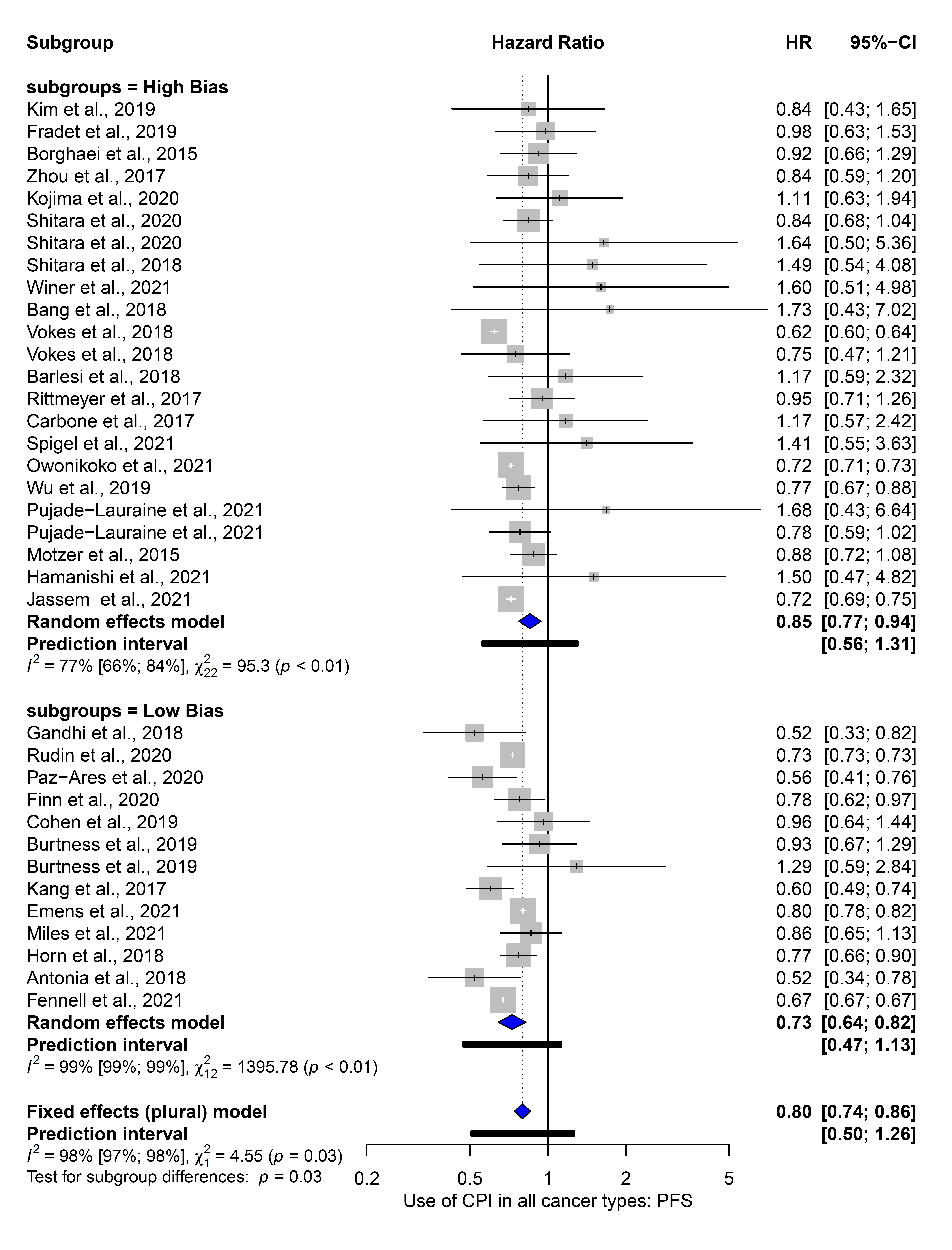


Figure S17 Subgroup meta-analysis of ICPI efficacy on PFS taken from studies deemed to have low vs high bias


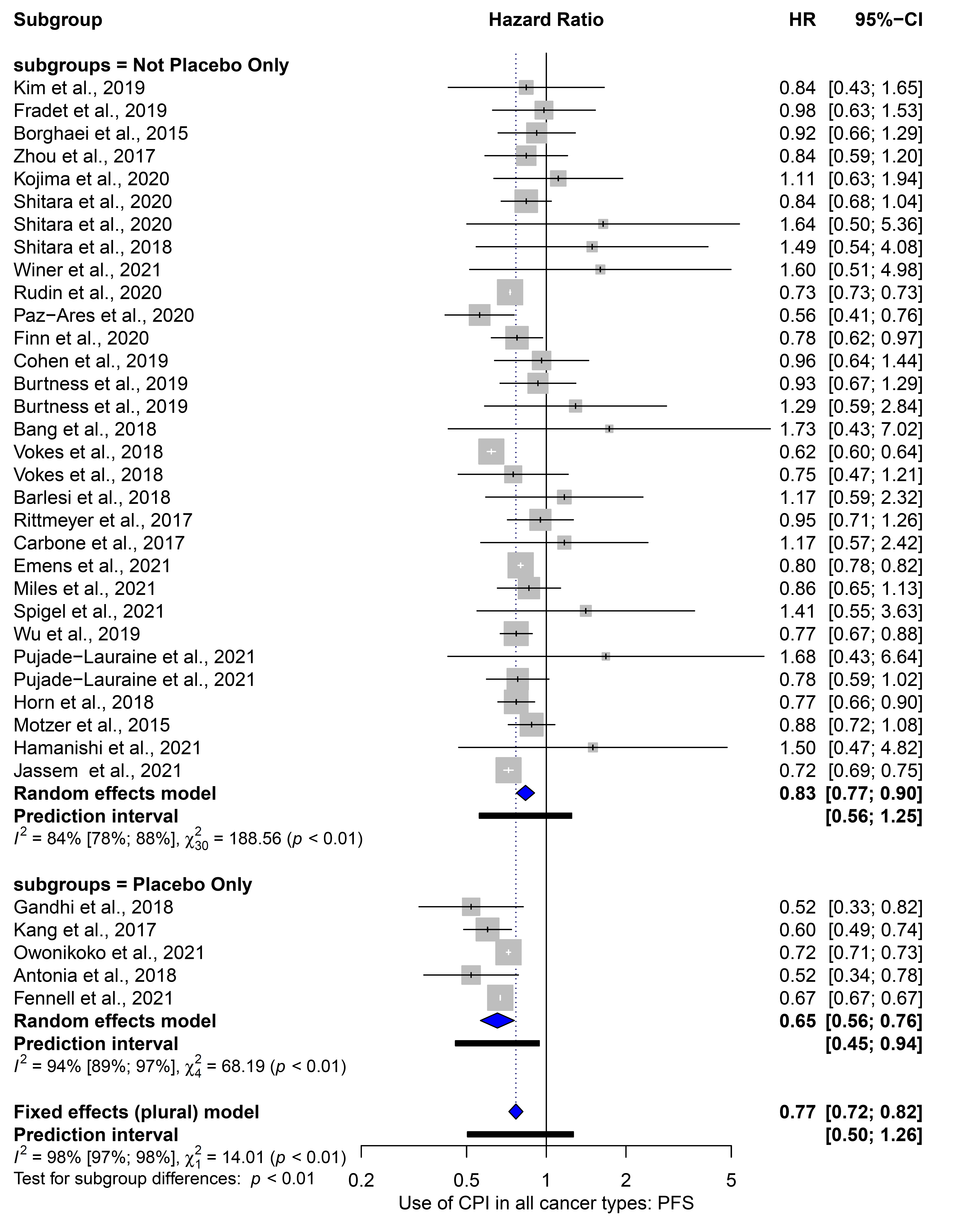


Figure S18 Subgroup meta-analysis of ICPI efficacy on PFS taken from studies with and without a placebo control arm.





Figure S19 Subgroup analysis showing risk of genitourinary side effects ICPI vs standard treatment/placebo





Figure S20 Subgroup analysis showing risk of gastrointestinal side effects ICPI vs standard treatment/placebo

*We present a summary graphic as to reduce space. Study by study break down is available on request to the authors (neil.ryan@ed.ac.uk).*





Figure S21 Subgroup analysis showing risk of dermatological side effects ICPI vs standard treatment/placebo


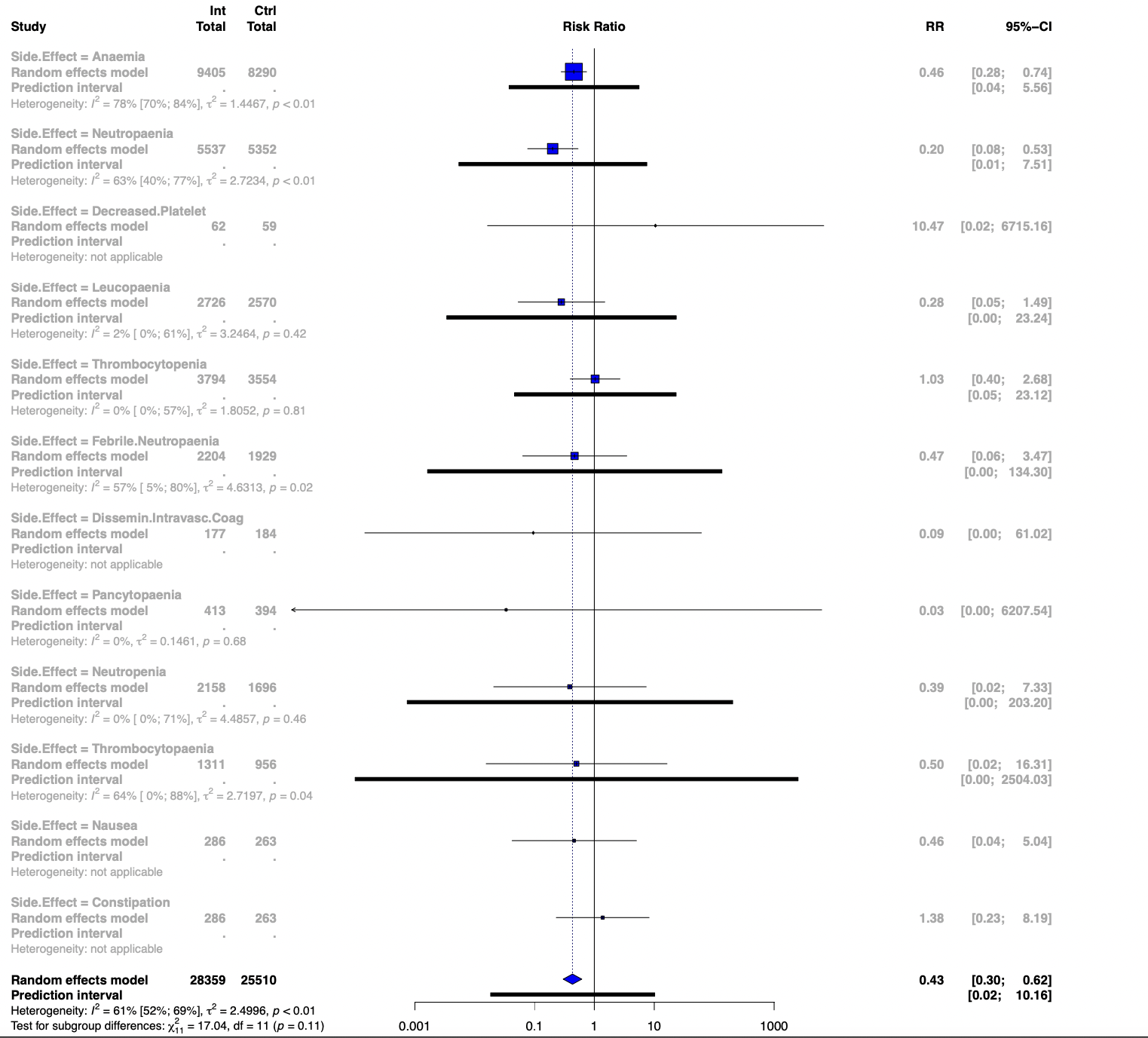


Figure S22 Subgroup analysis showing risk of haematological side effects ICPI vs standard treatment/placebo

*We present a summary graphic as to reduce space. Study by study break down is available on request to the authors (neil.ryan@ed.ac.uk).*


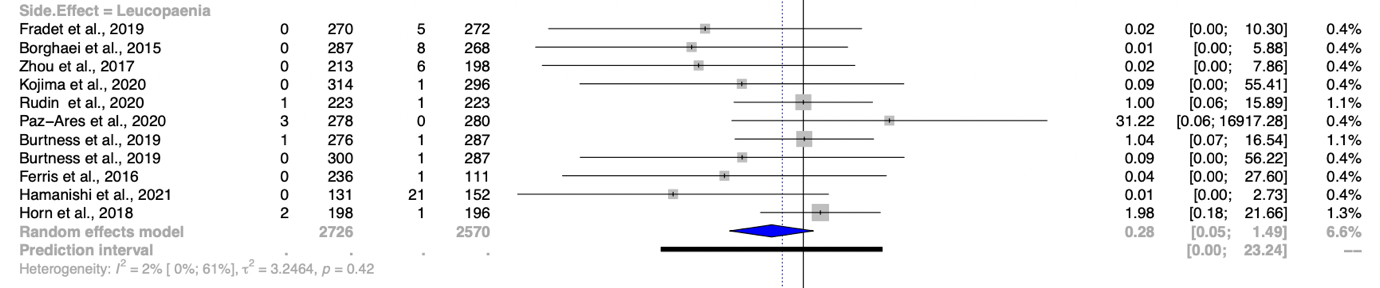

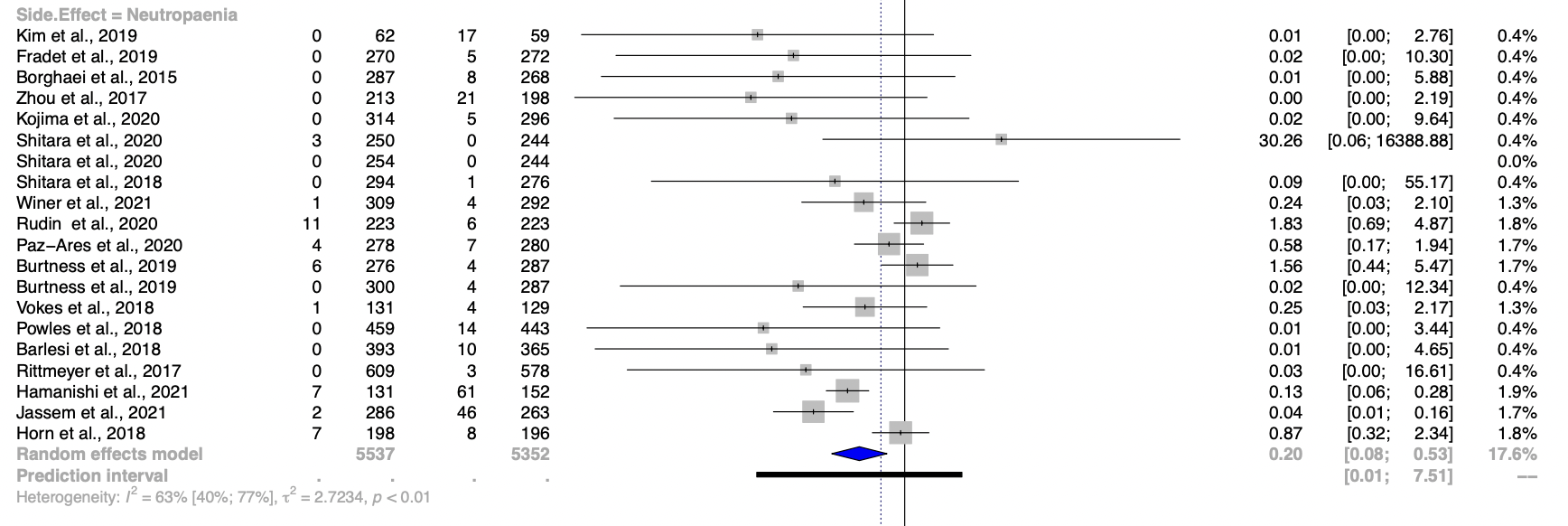

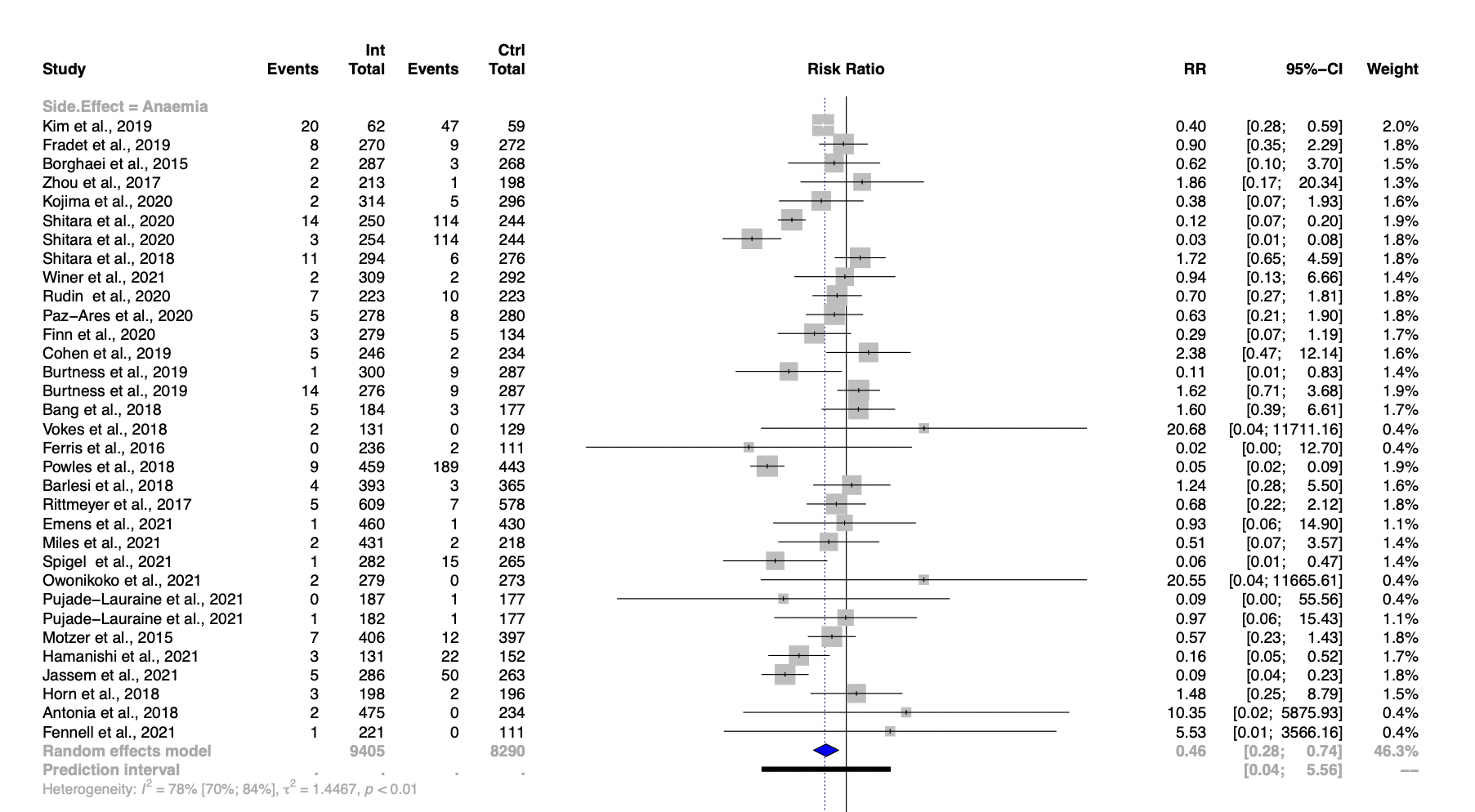


Figure S23 Subgroup analysis showing risk of anaemia with ICPI vs standard treatment/placebo

Figure S24 Subgroup analysis showing risk of neutropenia and leucopoenia with ICPI vs standard treatment/placebo


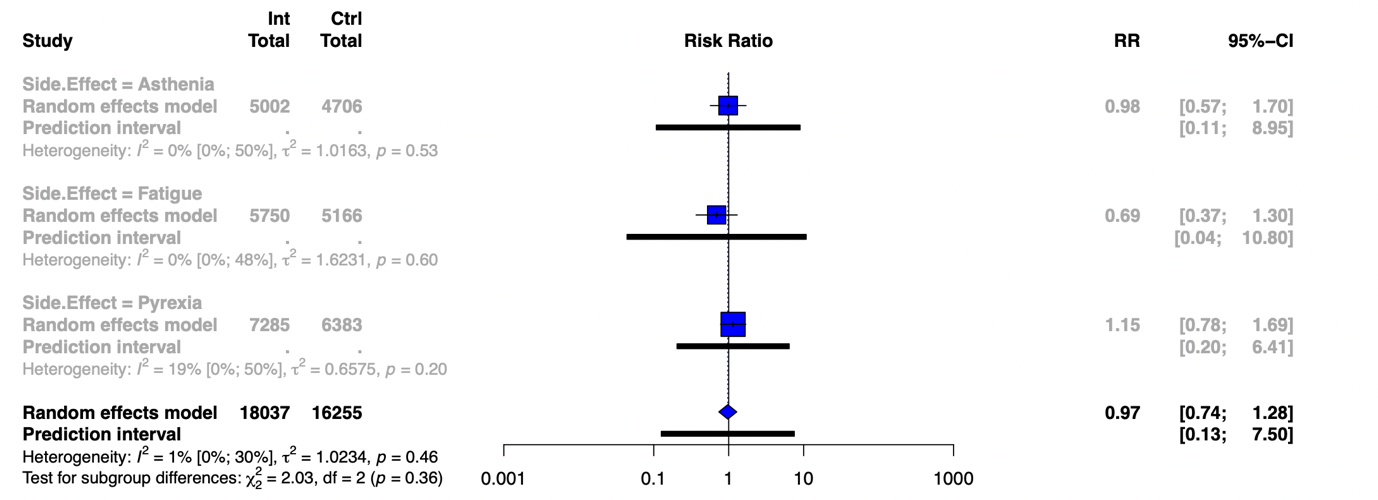


Figure S25 Subgroup analysis showing risk of neurological side effects ICPI vs standard treatment/placebo

*We present a summary graphic as to reduce space. Study by study break down is available on request to the authors (neil.ryan@ed.ac.uk).*


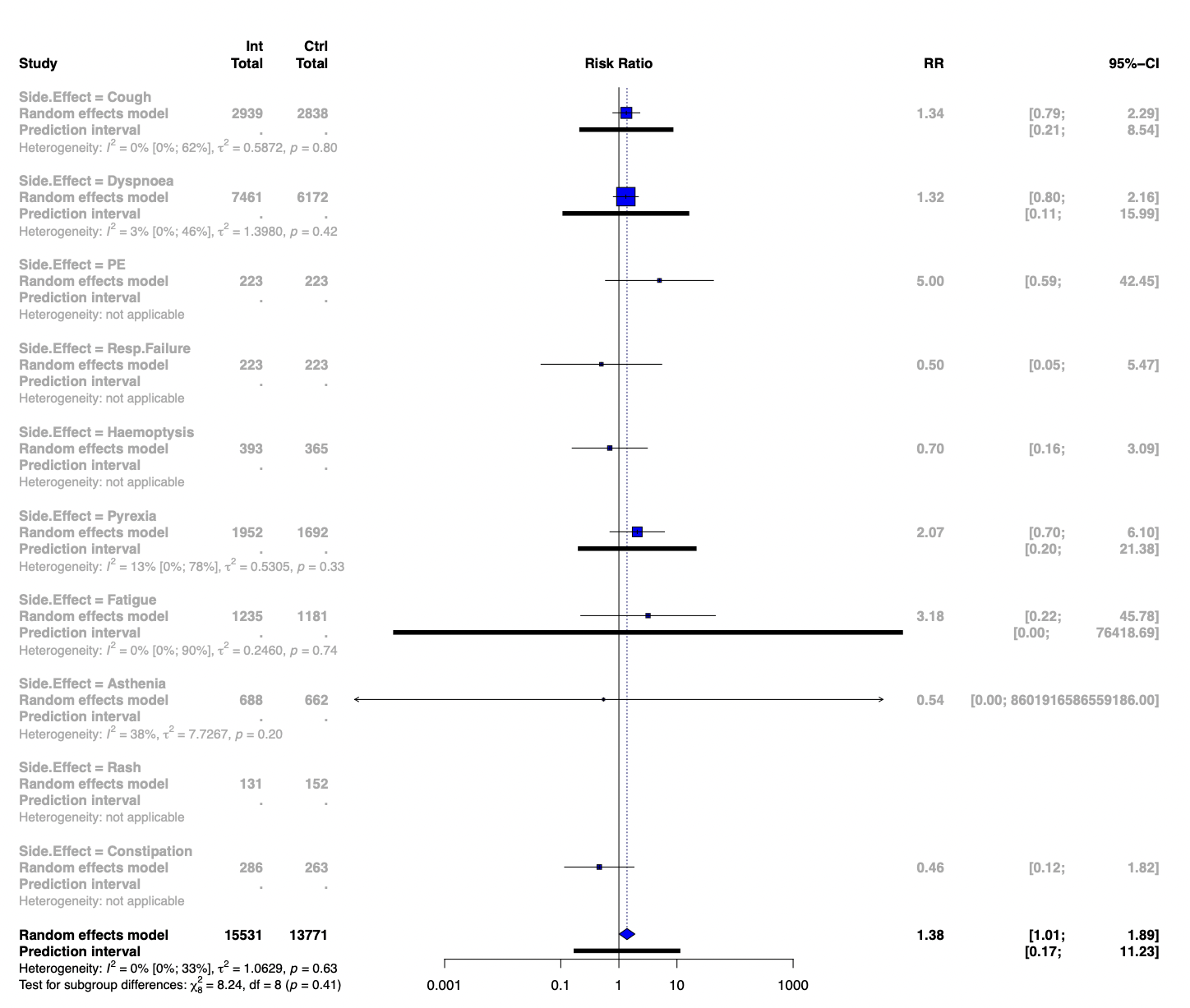


Figure S26 Subgroup analysis showing risk of other side effects ICPI vs standard treatment/placebo

*We present a summary graphic as to reduce space. Study by study break down is available on request to the authors (neil.ryan@ed.ac.uk).*

## 1.5 Subgroup analysis on PD-L1 expression

In total 25 studies reported PD-L1 expression. All bar two studies^6,8^ were deemed at high risk of bias (see Figure S26); subgroup analysis based on study bias found comparable OS benefit (high bias: HR 0.68 (95% CI 0.62 to 0.75) vs low bias HR 0.68 (95% CI 0.27 to 1.69)). The use of ICPIs in tumours with significant PD-L1 expression was associated with a significant improvement in OS (HR 0.68 (95% CI 0.62 to 0.74)) and PFS (HR 0.76 (95% CI 0.67 to 0.86)) regardless of tumour site; see Figures S27-28. Regarding specific tumour sites with significant PD-L1 expression, renal, ovarian, urothelial, mesothelium gastro-oesophageal and breast cancers had a trend to improved OS but failed to reach significance. However, low numbers of studies contributed to this analysis making meta-analysis fraught. Head and neck and Lung did show a significant improvement in OS. These data are summarised in Figure S29.

The effect of ICPI use in the advanced primary cancers and in the recurrence setting in cancers with a significant PD-L1 expression leads to an OS benefit with a HR 0.66 (95% CI 0.59 to 0.73) and HR 0.76 (95% CI 0.65 to 0.88) respectively however this did not reach significance (p=0.09) (Figure S30). The benefit in OS with higher PD-L1 expression was not dependent on the specific ICPI used (See Figure S31), with all bar Avelumab reporting similar OS benefit. Only one study reported PD-L1 status in the Avelumab subgroup, and this result failed to reach significance however demonstrated a positive trend. Only two studies compared an ICPI against a placebo arm and therefore meta-analysis was of limited value; these data are summarised in Figure S32.


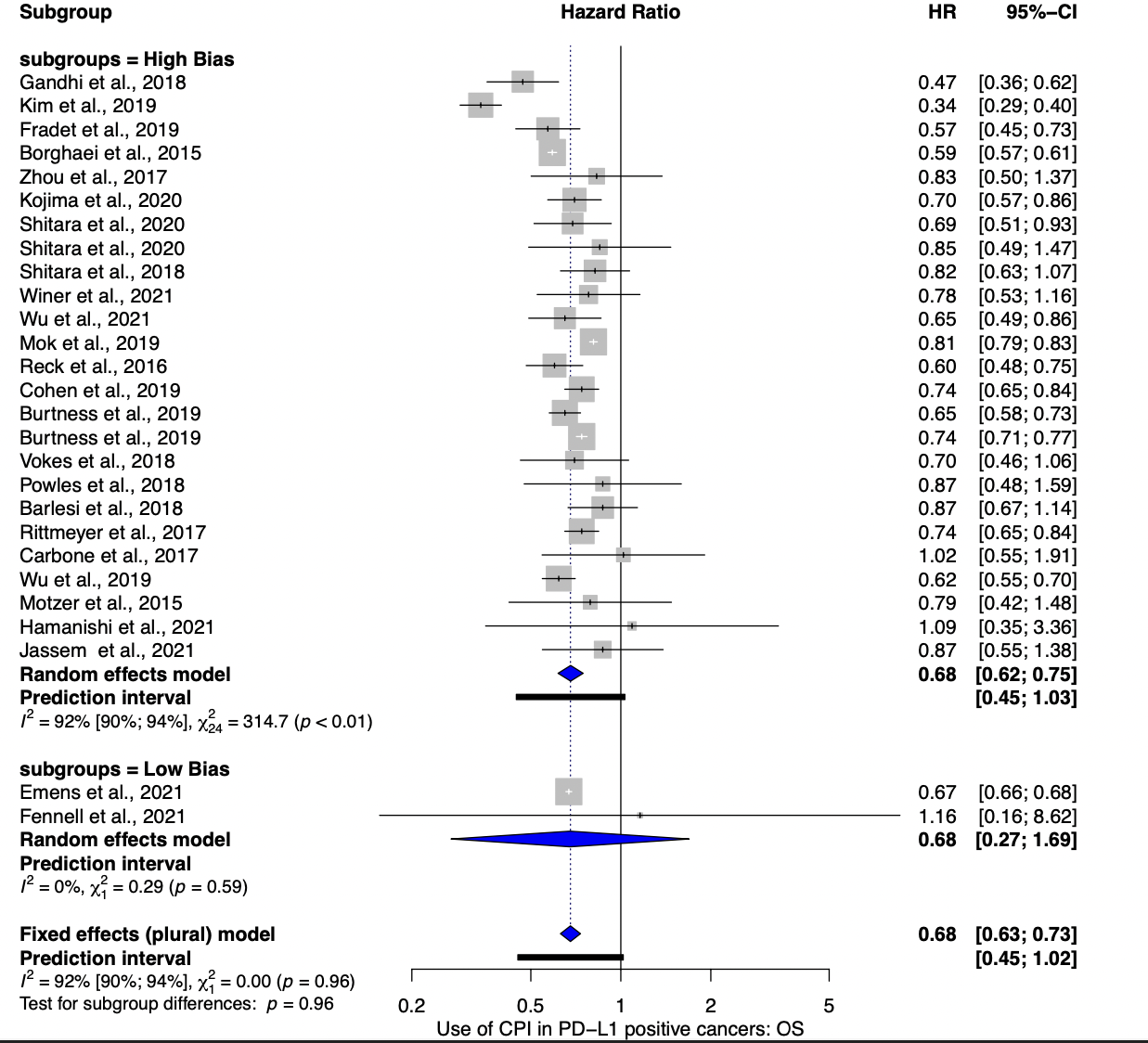


Figure S27 Subgroup meta-analysis of ICPI efficacy on OS taken from studies in which PD-L1 expression was deemed significant by the authors grouped by study bias


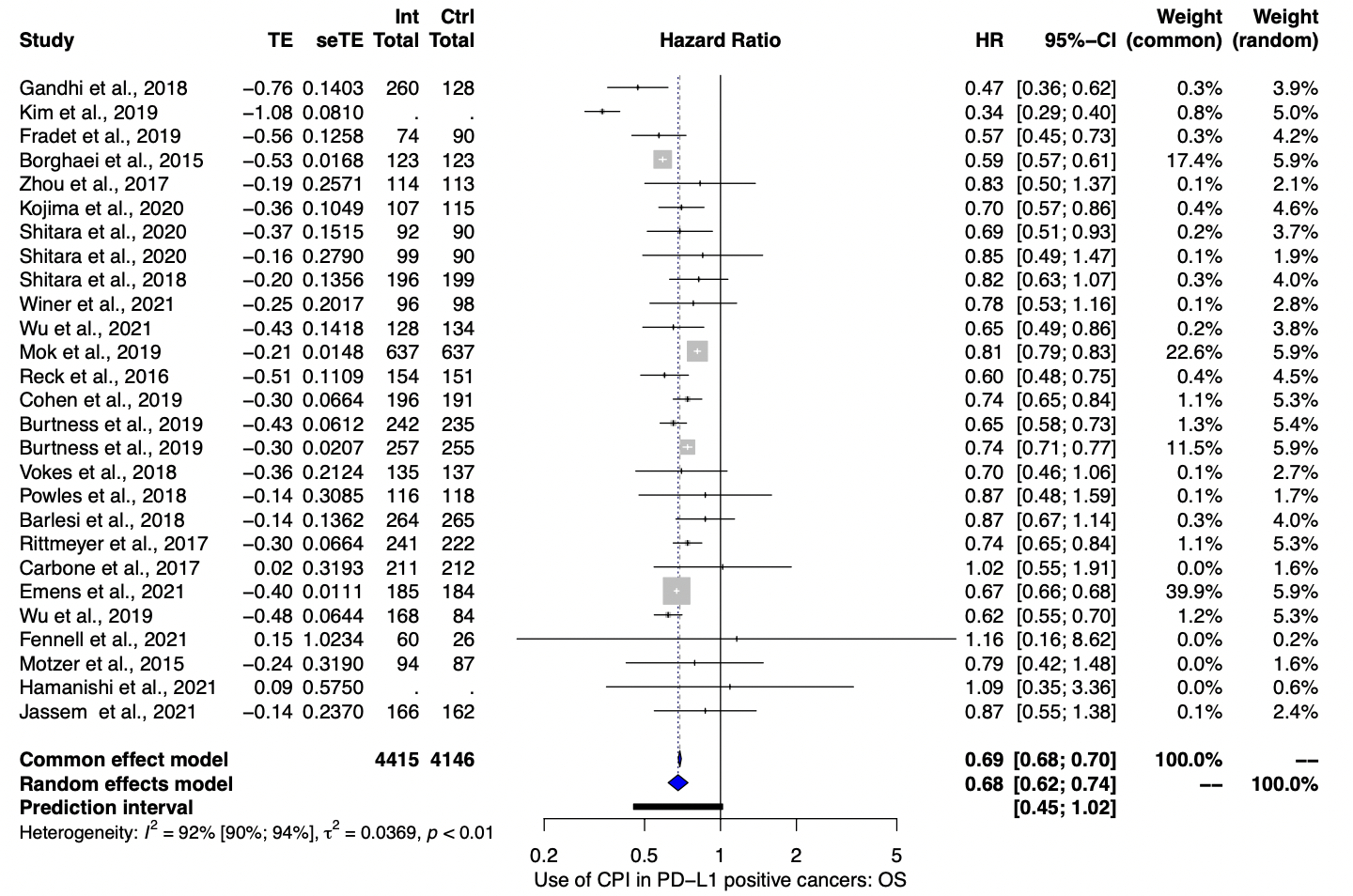


Figure S28 Subgroup meta-analysis of ICPI efficacy on OS taken from studies in which PD-L1 expression was deemed significant by the authors


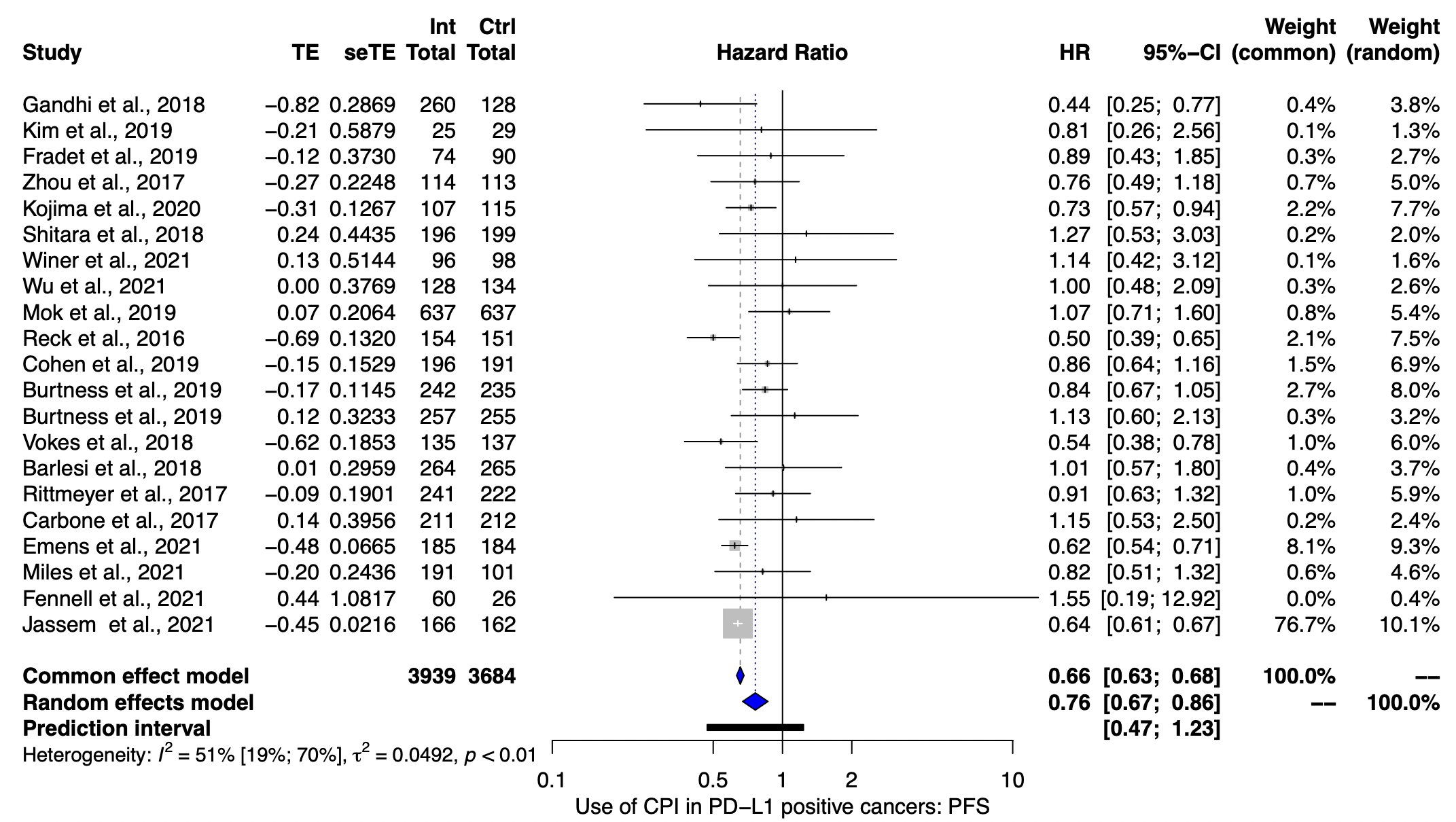


Figure S29 Subgroup meta-analysis of ICPI efficacy on PFS taken from studies in which PD-L1 expression was deemed significant by the authors


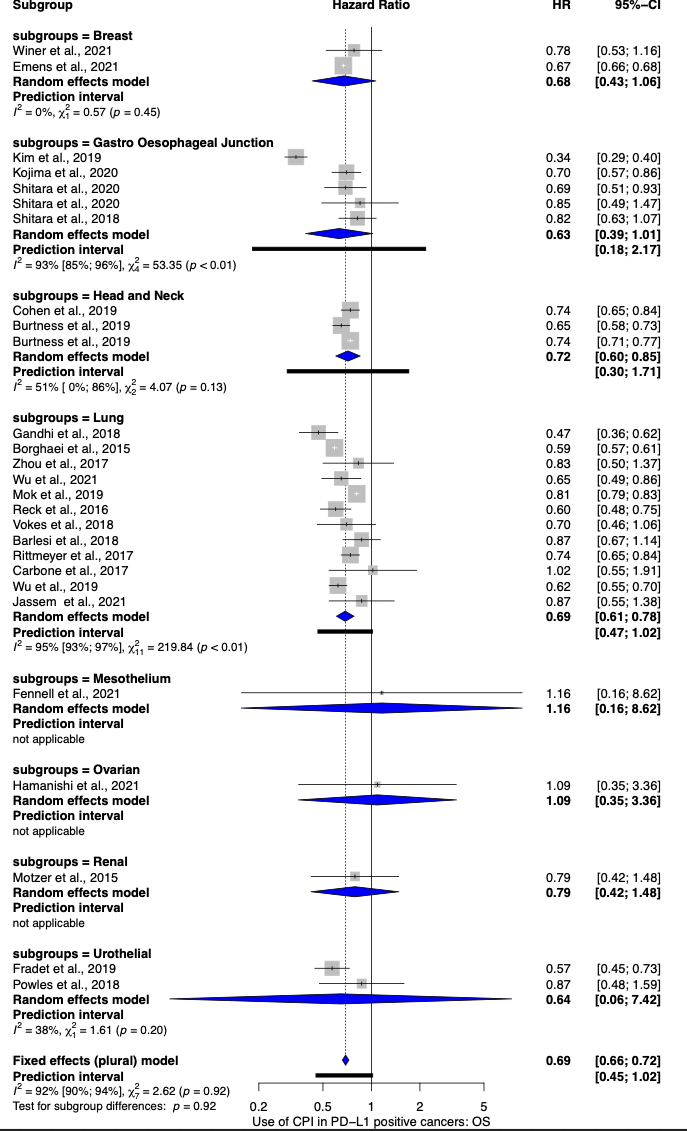


Figure S30 Subgroup meta-analysis of ICPI efficacy on OS displayed by cancer site taken from studies in which PD-L1 expression was deemed significant by the authors


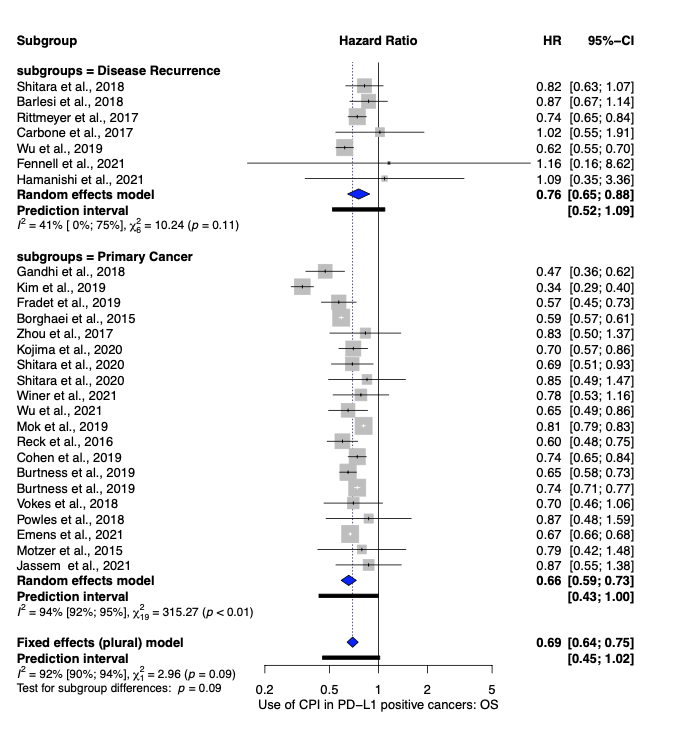


Figure S31 Subgroup meta-analysis of ICPI efficacy on OS displayed by primary cancer and disease recurrence from studies in which PD-L1 expression was deemed significant by the authors


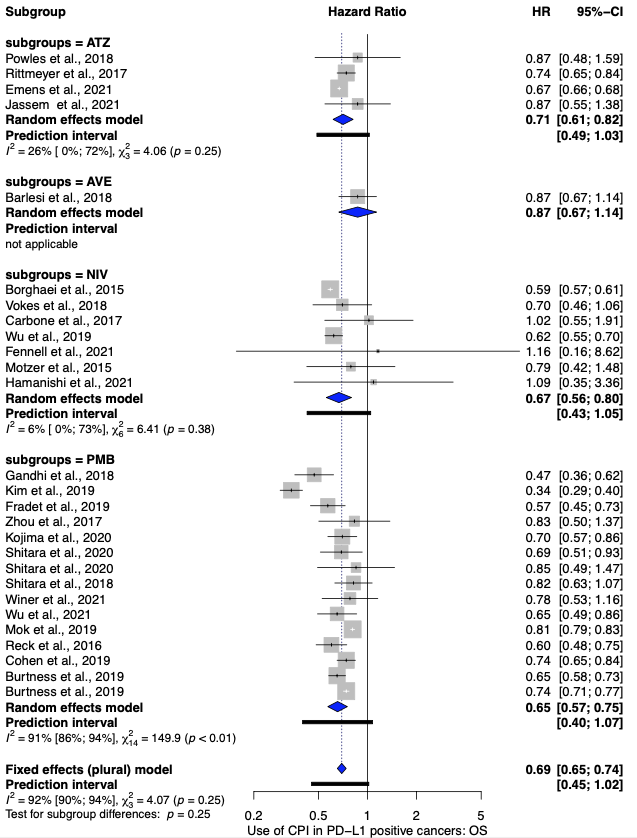


Figure S31 Subgroup meta-analysis comparting the OS in grouped by studies that used different ICPI agents from studies in which PD-L1 expression was deemed significant by the authors


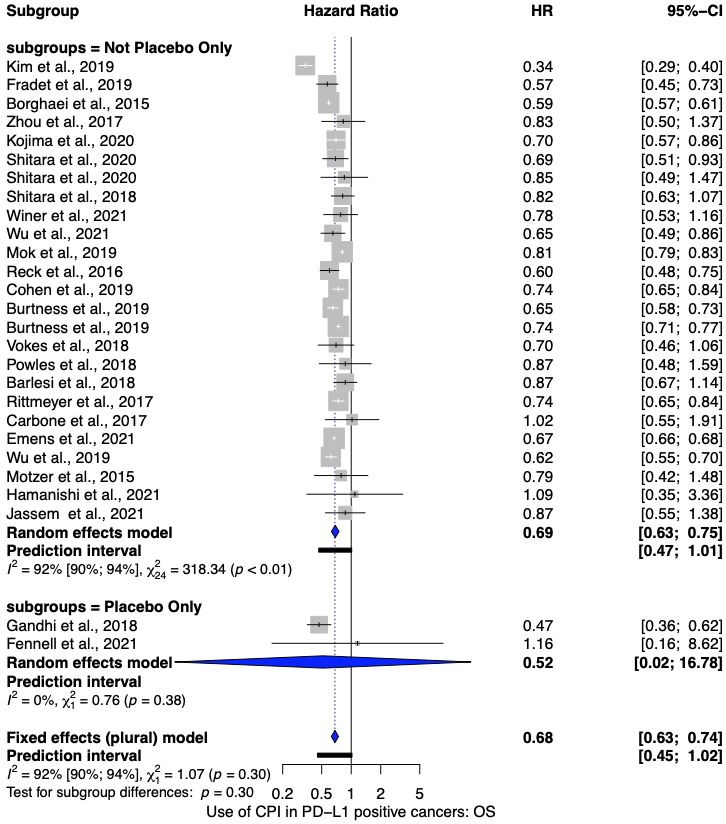


Figure S32 Subgroup meta-analysis comparting the OS in grouped by studies those with a placebo and non-placebo arms from studies in which PD-L1 expression was deemed significant by the authors

## Reference:

1. Higgins JPT, Thompson SG. Quantifying heterogeneity in a meta‐analysis. *Stat Med*. 2002;21(11):1539-1558. doi:10.1002/sim.1186

2. Simonsohn U, Nelson LD, Simmons JP. p -Curve and Effect Size: Correcting for Publication Bias Using Only Significant Results. *Perspect Psychol Sci*. 2014;9(6):666-681. doi:10.1177/1745691614553988

3. Egger M, Smith GD, Schneider M, Minder C. Bias in meta-analysis detected by a simple, graphical test. *Bmj*. 1997;315(7109):629. doi:10.1136/bmj.315.7109.629

4. Vokes EE, Ready N, Felip E, et al. Nivolumab versus docetaxel in previously treated advanced non-small-cell lung cancer (CheckMate 017 and CheckMate 057): 3-year update and outcomes in patients with liver metastases. *Ann Oncol*. 2018;29(4):959-965. doi:10.1093/annonc/mdy041

5. Burtness B, Harrington KJ, Greil R, et al. Pembrolizumab alone or with chemotherapy versus cetuximab with chemotherapy for recurrent or metastatic squamous cell carcinoma of the head and neck (KEYNOTE-048): a randomised, open-label, phase 3 study. *Lancet Lond Engl*. 2019;394(10212):1915-1928. doi:10.1016/s0140-6736(19)32591-7

6. Fennell DA, Ewings S, Ottensmeier C, et al. Nivolumab versus placebo in patients with relapsed malignant mesothelioma (CONFIRM): a multicentre, double-blind, randomised, phase 3 trial. *Lancet Oncol*. 2021;22(11):1530-1540. doi:10.1016/s1470-2045(21)00471-x

7. Rudin CM, Awad MM, Navarro A, et al. Pembrolizumab or Placebo Plus Etoposide and Platinum as First-Line Therapy for Extensive-Stage Small-Cell Lung Cancer: Randomized, Double-Blind, Phase III KEYNOTE-604 Study. *J Clin Oncol*. 2020;38(21):2369-2379. doi:10.1200/jco.20.00793

8. Emens LA, Adams S, Barrios CH, et al. First-line atezolizumab plus nab-paclitaxel for unresectable, locally advanced, or metastatic triple-negative breast cancer: IMpassion130 final overall survival analysis. *Ann Oncol*. 2021;32(8):983-993. doi:10.1016/j.annonc.2021.05.355
